# Survival benefits of different immunotherapies for hepatocellular carcinoma: a meta-analysis highlighting age, gender, etiology, and tumor burden

**DOI:** 10.3389/fimmu.2025.1713151

**Published:** 2025-12-10

**Authors:** Ming-Cheng Guan, Na Li, Qian Ding, Di Sun, Hao Li, Mei Yang, Lei Hong, Hong Zhu

**Affiliations:** 1 Department of Medical Oncology, The First Affiliated Hospital of Soochow University, Suzhou, Jiangsu, China; 2 Department of Medical Oncology, Affiliated Hospital of Jiangnan University, Wuxi, Jiangsu, China; 3 Advanced Molecular Pathology Institute of Soochow University and SANO, Suzhou, Jiangsu, China; 4 Institute of Clinical Medicine Research, Suzhou Hospital, Affiliated Hospital of Medical School, Nanjing University, Suzhou Science and Technology Town Hospital, Suzhou, Jiangsu, China

**Keywords:** hepatocellular carcinoma, immunotherapy, subgroup analysis, age, gender, meta-analysis, immune checkpoint inhibitor

## Abstract

**Background:**

The heterogeneous efficacy of immunotherapy in hepatocellular carcinoma (HCC) remains unclear. We evaluated efficacy and safety of various immunotherapeutic regimens—including immune checkpoint inhibitor (ICI) monotherapy, dual ICIs, and ICI plus targeted therapy—for unresectable HCC, to identify patient subgroups that benefit most.

**Methods:**

Randomized clinical trial evaluating immunotherapy as first-line treatment for unresectable HCC versus tyrosine kinase inhibitors (TKIs) were systematically searched. Pooled hazard ratios (HRs) for overall survival (OS) and progression-free survival (PFS), and odds ratios (ORs) for objective response rate (ORR), disease control rate (DCR), and treatment-related adverse events (TRAEs) were calculated.

**Results:**

Twelve trials were included. Immunotherapy significantly improved OS (HR = 0.77 [0.71-0.83]) and PFS (HR = 0.73 [0.63-0.84]) versus TKIs. ICI plus targeted therapy showed the greatest benefit, reducing mortality by 27% and progression risk by 37%. Subgroup analyses revealed patients aged ≥65 years and male patients derived substantial OS and PFS benefits, particularly from ICI plus targeted therapy, whereas younger patients (<65 years) benefited more from dual ICIs. Additional favorable subgroups included Asian patients, HBV-positive patients, those with poor performance status, macrovascular invasion and/or extrahepatic spread, and Barcelona Clinic Liver Cancer stage C. Notably, female patients showed no significant OS improvement across any regimen. Moreover, non-Asian patients, those with hepatitis C, BCLC stage B, or AFP <400 ng/mL derived limited immunotherapy benefit across regimens.

**Conclusions:**

Immunotherapy improves survival in unresectable HCC, with differential subgroup benefits highlighting the necessity for personalized strategies.

**Systematic Review Registration:**

https://www.crd.york.ac.uk/prospero/, identifier CRD42025635108.

## Introduction

Hepatocellular carcinoma (HCC) represents a significant global health burden, ranking among the leading causes of cancer-related mortality worldwide (1). The incidence is particularly high in Asia, where hepatitis B virus (HBV) infection remains the predominant etiological factor, contributing to a substantial proportion of HCC cases (2). Notably, HCC exhibits a marked male predominance, and the majority of patients are diagnosed at an advanced stage, where systemic therapy is the primary treatment option (2, 3). Historically, tyrosine kinase inhibitors (TKIs) such as Sorafenib and Lenvatinib have been the cornerstone of first-line systemic treatment (4). However, these targeted agents offer only modest survival benefits, underscoring the need for more effective therapeutic strategies. The advent of immune checkpoint inhibitors (ICIs) has transformed the treatment landscape for multiple malignancies, including HCC (5–7). Since the pivotal IMbrave150 trial in 2020 (8), accumulating evidence has demonstrated the clinical efficacy of ICIs, either as monotherapy or in combination with targeted agents, in improving survival outcomes in HCC patients (9).

Despite the growing body of evidence supporting immunotherapy in HCC, several critical knowledge gaps remain unaddressed. Previous meta-analyses have indicated that the benefits of ICIs may be more pronounced in Asian populations, HBV-associated HCC, and male patients (10–14). However, other yet-unidentified subgroups may also derive significant benefit from immunotherapy; these meta-analyses included few randomized controlled trials (RCTs) and/or many retrospective studies, limiting their statistical power and generalizability (15). Moreover, prior analyses have not adequately compared the efficacy and safety of different immunotherapeutic strategies, including ICI monotherapy (I), dual ICIs (I+I), and ICI plus targeted therapy (T+I) (16). Additionally, the impact of patient-specific factors, such as baseline clinical characteristics, on immunotherapy efficacy has not been systematically explored (17).

This meta-analysis seeks to address these gaps by conducting a comprehensive evaluation of the efficacy and safety of various immunotherapeutic regimens for unresectable HCC. The exclusion of retrospective studies can enhance the quality of the studies included and the results of the meta-analysis (18), so our study incorporates newly published randomized controlled trials (RCTs) and conference data, ensuring a more updated and robust synthesis of available evidence. Moreover, we employ subgroup analyses based on different immunotherapeutic strategies (e.g., combination therapy versus monotherapy, as well as ICI monotherapy versus dual ICIs versus ICI plus targeted therapy) and key clinical parameters (e.g., geographic region, age, gender, etiology, disease burden, and biomarker status), to delineate patient populations that derive the greatest benefit from immunotherapy. By leveraging a rigorous methodological framework, this study aims to refine current treatment paradigms and provide clinically relevant insights that can inform personalized therapeutic decision-making for HCC patients.

## Materials and methods

### Search strategies and study selection

This meta-analysis followed the guidelines of the Preferred Reporting Items for Systematic Reviews and Meta-Analyses (PRISMA) and was registered in the International Prospective Register of Systematic Reviews (PROSPERO, CRD42025635108). Two independent researchers (Ming-Cheng Guan and Na Li) systematically searched PubMed, Medline, and Embase for relevant studies. To ensure comprehensive data collection, additional searches were conducted in ClinicalTrials.gov and within conference abstracts from the American Society of Clinical Oncology (ASCO), the European Society for Medical Oncology (ESMO), and the Chinese Society of Clinical Oncology (CSCO). The search period spanned from the inception of each database or conference to February 20, 2025. The predefined Medical Subject Headings (MeSH) terms included “immunotherapy,” “immune therapy,” “immune checkpoint inhibitor,” “immune checkpoint blockade,” “CTLA-4 inhibitor,” “PD-1 inhibitor,” “PD-L1 inhibitor,” “hepatocellular carcinoma,” and “liver cancer.” These terms were combined in various ways, with “clinical trial” used as a qualifier.

To evaluate how immunotherapy influences the survival outcomes of HCC and to minimize inter-study heterogeneity, the inclusion criteria were carefully defined as follows: phase II or III clinical trials; patients diagnosed with HCC based on pathological confirmation or characteristic imaging features; ICI therapies used as first-line treatment; targeted therapy as the comparator; and available data on overall survival (OS) or progression-free survival (PFS). Studies that employed immunotherapy or placebo as controls, or those investigating locoregional therapy as an intervention, were excluded. In cases where multiple publications reported data from the same trial, the most recent version was selected. Only studies published in English or Chinese were considered.

Based on these criteria, three reviewers (Ming-Cheng Guan, Na Li, and Qian Ding) independently screened titles and abstracts to remove irrelevant records. Full-text reviews were then conducted to identify studies meeting the eligibility criteria. Ultimately, the selected studies were included in the meta-analysis.

### Data collection

Data extraction was carried out independently by two investigators (Ming-Cheng Guan and Na Li), following a predefined data collection framework. Any disagreements were resolved through discussion with a third reviewer (Qian Ding). The extracted variables included trial ID, authorship, publication year, registration number, treatment setting, trial phase, enrolled patient count, treatment regimens, and outcome measures such as OS, PFS, objective response rate (ORR), disease control rate (DCR), and treatment-related adverse events (TRAE) in both the overall population and relevant subgroups. When hazard ratios (HRs) and 95% confidence intervals (CIs) were not explicitly provided in the text, values were digitized using GetData Graph Digitizer (19). Additionally, clinical context for each study was documented. To ensure data accuracy, the final dataset was cross-verified by multiple investigators.

### Risk of bias assessment

The risk of bias for RCTs included in this analysis was evaluated using the Cochrane Risk of Bias Tool within Review Manager 5.4. Two independent investigators assessed methodological quality across six categories: selection bias, performance bias, detection bias, attrition bias, reporting bias, and other sources of potential bias. Each domain was classified as having a low, high, or unclear risk after a detailed examination of the study methodology and supplementary materials. In cases of discrepancies, the reviewers (Ming-Cheng Guan and Di Sun) consulted existing literature and sought arbitration from a third reviewer to reach a consensus.

### Statistical analysis

Statistical analyses were conducted using the “meta” package in R (20, 21). The pooled HRs with 95% CIs for OS and PFS, as well as pooled odds ratios (ORs) with 95% CIs for ORR, DCR, and TRAE, were calculated. A *P*-value < 0.05 was considered statistically significant. If the 95% CI did not cross or include 1, the result was deemed significant (22). Study heterogeneity was evaluated using the I² statistic, with I² > 50% or *P* < 0.1 indicating substantial heterogeneity, necessitating the use of a random-effects model. Otherwise, a fixed-effects model was applied. Publication bias was assessed using Begg’s and Egger’s tests, where a *P*-value > 0.05 suggested no significant bias. To further explore potential biases, funnel plots and Baujat plots were generated. A sensitivity analysis was performed by sequentially removing individual studies to determine their influence on the overall pooled HR and OR.

## Results

### Study characteristics

**Figure 1:**
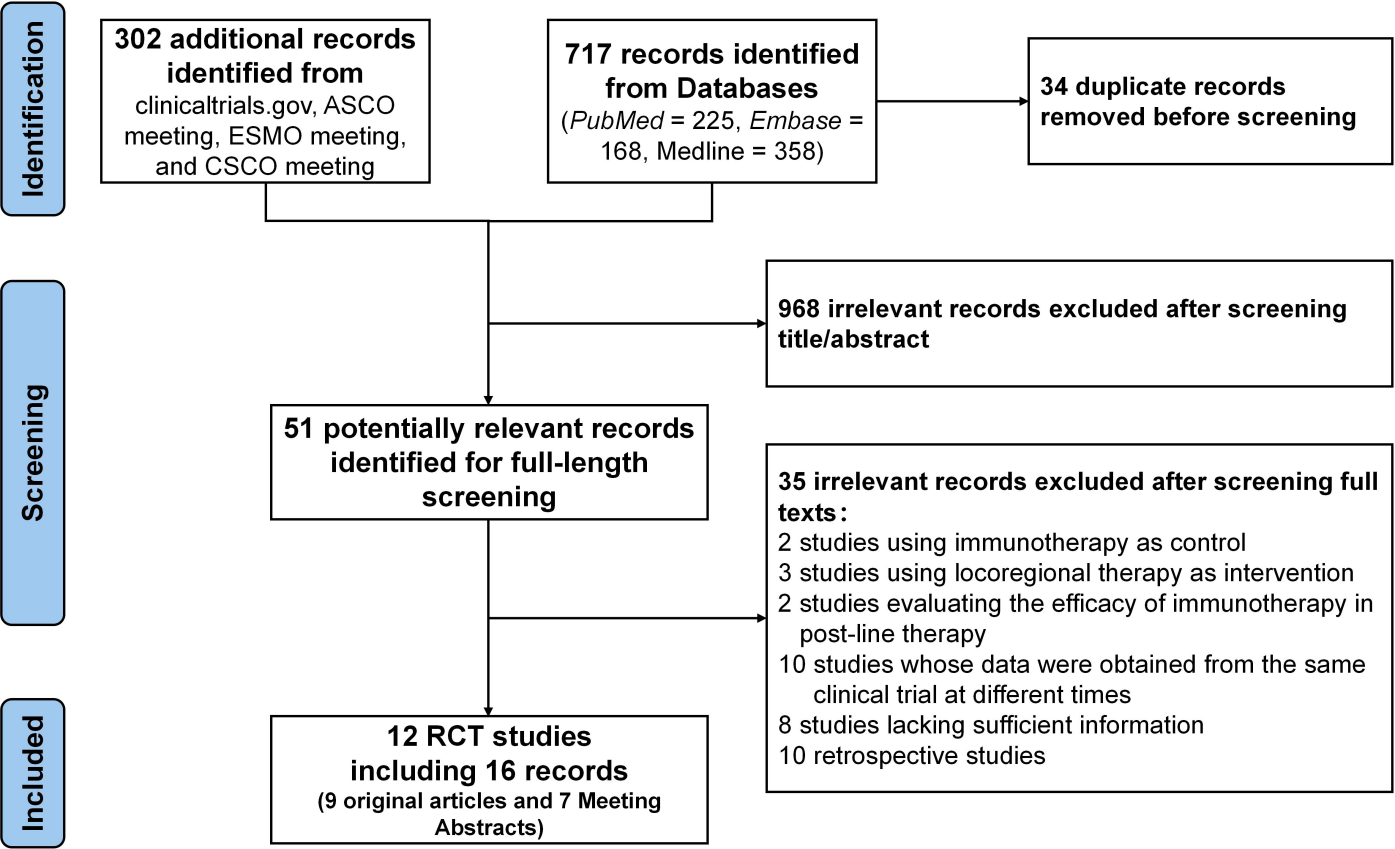
Flow diagram of trials selection.

The study flowchart is illustrated in **Figure 1**. A total of 16 relevant publications were identified, corresponding to 12 distinct RCTs (23–38). Several trials had multiple related reports; specifically, the data from CARES-310 (24) and HIMALAYA (32) were partly obtained from the most recent conference reports, with PFS data for HIMALAYA extracted from its earliest published study (31). The ORR data for HCC caused by different etiologies in HIMALAYA were derived from conference reports (33). In such cases, data from the most comprehensive and recent publication were used for meta-analysis.

A total of 7,635 patients from the 12 eligible trials were included in the quantitative synthesis. All trials evaluated the efficacy and safety of immunotherapies as first-line treatments, with TKIs (Sorafenib or Lenvatinib) as control. Eight trials (CARES-310 (23, 24), COSMIC-312 (27), IMbrave150 (25), LEAP-002 (28), ORIENT-32 (26), APOLLO (35), HEPATORCH (38), and SCT-I10A-C301 (37)) assessed the efficacy of ICI plus targeted therapy versus TKIs, while two trials (CheckMate 459 (29) and RATIONALE-301 (34)) evaluated ICI monotherapy against TKIs. One trial (CheckMate 9DW) (36) examined dual ICIs versus TKIs, and another (HIMALAYA) (30–33) investigated both dual ICIs and monotherapy in comparison to TKIs. Of the 12 trials, eight trials were published as full-text articles, whereas the remaining four were available as conference abstracts. The baseline characteristics of included studies are summarized in **Table 1**.

**Table 1:** Main characteristics of each included trials comparing immunotherapy with TKI.

Trial ID	Author year	Registration no.	Arm (immunotherapy vs. TKI)	Numbers of parients	Median OS, months	HR for OS (95%CI)	Median PFS, months	HR for PFS (95%CI)
CARES-310	Qin 2023	NCT03764293	Camrelizumab plus Rivoceranib vs. Sorafenib	272 vs. 271	23.8 (20.6, 27.2) vs. 15.2 (13.2, 18.5)	0.64 (0.52, 0.79)	5.6 (5.5, 7.4) vs. 3.7 (3.1, 3.7)	0.54 (0.44, 0.67)
CheckMate 459	Yau 2022	NCT02576509	Nivolumab vs. Sorafenib	371 vs. 372	16.4 (13.9, 18.4) vs. 14.7 (11.9, 17.2)	0.85 (0.72, 1.02)	3.7 (3.1, 3.9) vs. 3.8 (3.7, 4.5)	0.93 (0.79, 1.10)
COSMIC-312	Yau 2024	NCT03755791	Cabozantinib plus Atezolizumab vs. Sorafenib	432 vs. 217	16.5 (14.5, 18.7) vs. 15.5 (12.2, 20.0)	0.98 (0.78, 1.22)†	6.9 (5.7, 8.2) vs. 4.3 (2.9, 6.1)	0.74 (0.60, 0.91)‡
HIMALAYA	Sangro 2024	NCT03298451	Tremelimumab plus Durvalumab (STRIDE) vs. Durvalumab vs. Sorafenib	393 vs. 389 vs. 389	16.4 (14.2, 19.6) vs. 16.6 (14.1-19.1) vs.13.8 (12.3, 16.1)	0.76 (0.65, 0.89);0.86 (0.74, 1.01)	3.78 (3.68, 5.32) vs. 3.65 (3.19, 3.75) vs. 4.07 (3.75, 5.49)	0.90 (0.77, 1.05); 1.02 (0.88, 1.19)
IMbrave150	Cheng 2022	NCT03434379	Atezolizumab plus Bevacizumab vs. Sorafenib	336 vs. 165	19.2 (17.0, 23.7) vs. 13.4 (11.4, 16.9)	0.66 (0.52, 0.85)	6.9 (5.7, 8.6) vs. 4.3 (4.0, 5.6)	0.65 (0.53, 0.81)
LEAP-002	Llovet 2023	NCT03713593	Pembrolizumab plus Lenvatinib vs. Lenvatinib plus Placebo	395 vs. 399	21.2 (19.0, 23.6) vs. 19.0 (17.2, 21.7)	0.84 (0.71, 1.00)	8.2 (6.3, 8.3) vs. 8.1 (6.3, 8.3)	0.83 (0.71, 0.98)
ORIENT-32	Ren 2021	NCT03794440	Sintilimab plus Bevacizumab Biosimilar (IBI305) vs. Sorafenib	380 vs. 191	NR (NR, NR) vs. 10.4 (8.5, NR)	0.57 (0.43, 0.75)	4.6 (4.1, 5.7) vs. 2.8 (2.7, 3.2)	0.56 (0.46, 0.70
RATIONALE-301	Qin 2023	NCT03412773	Tislelizumab vs. Sorafenib	342 vs. 332	15.9 (13.2, 19.7) vs. 14.1 (12.6, 17.4)	0.85 (0.71, 1.02)	2.1 (2.1, 3.5) vs. 3.4 (2.2, 4.1)	1.11(0.92, 1.33)
APOLLO	Zhou 2024	NCT04344158	Anlotinib plus Penpulimab vs. Sorafenib	433 vs. 216	16.5 (14.7, 19.7) vs. 13.2 (9.7, 16.9)	0.69 (0.55, 0.86)ξ	6.9 (5.7, 8.0) vs. 2.8 (2.7, 4.1)	0.53 (0.42, 0.68) †
CheckMate 9DW	Decaens 2024	NCT04039607	Nivolumab plus Ipilimumab vs. Lenvatinib or Sorafenib	335 vs. 333	23.7 (18.8, 29.4) vs. 20.6 (17.5, 22.5)	0.79 (0.65, 0.96)	7.5 (6.3, 9.2) vs. 7.5 (7.2, 9.2)	0.72 (0.60, 0.86)
HEPATORCH	Fan 2024	NCT04723004	Toripalimab plus Bevacizumab vs. Sorafenib	162 vs. 164	20.0 (15.3, 23.4) vs. 14.5(11.4, 18.8)	0.76 (0.58, 0.99)	5.8 (4.6, 7.2) vs. 4.0 (2.8, 4.2)	0.69 (0.53, 0.91)
SCT-I10A-C301	Xu 2024	NCT04560894	SCT-I10A plus Bevacizumab Biosimilar (SCT510) vs. Sorafenib	230 vs. 116	22.1 (18.6, NR) vs. 14.2 (10.2, 15.8)	0.60 (0.44, 0.8)	7.1 (6.1, 8.4) vs. 2.9 (2.8, 4.1)	0.50 (0.38, 0.65)

†Converted based on 96% CI; ‡ Converted based on 99% CI; ξ Converted based on 98.8% CI.

All control arms used TKIs, including sorafenib or lenvatinib, as standard first-line treatments for unresectable hepatocellular carcinoma.

CI, confidence interval; HR, hazard ratio; NR, not reached; OS, overall survival; PFS, progression-free survival; TKI, tyrosine kinase inhibitors.

### The risk of bias of the included studies

The methodological quality of the included studies was assessed as high, low, or unclear risk of bias. Overall, the risk of bias was low; however, 11 of the 12 trials were open-label, leading to a high risk of performance bias. Additionally, four trials were published as conference abstracts, resulting in an increased level of uncertainty due to incomplete disclosure of study design details. The detailed risk of bias assessment is presented in **Supplementary Figure 1**.

### Efficacy and safety of immunotherapy in the whole population

**Figure 2:**
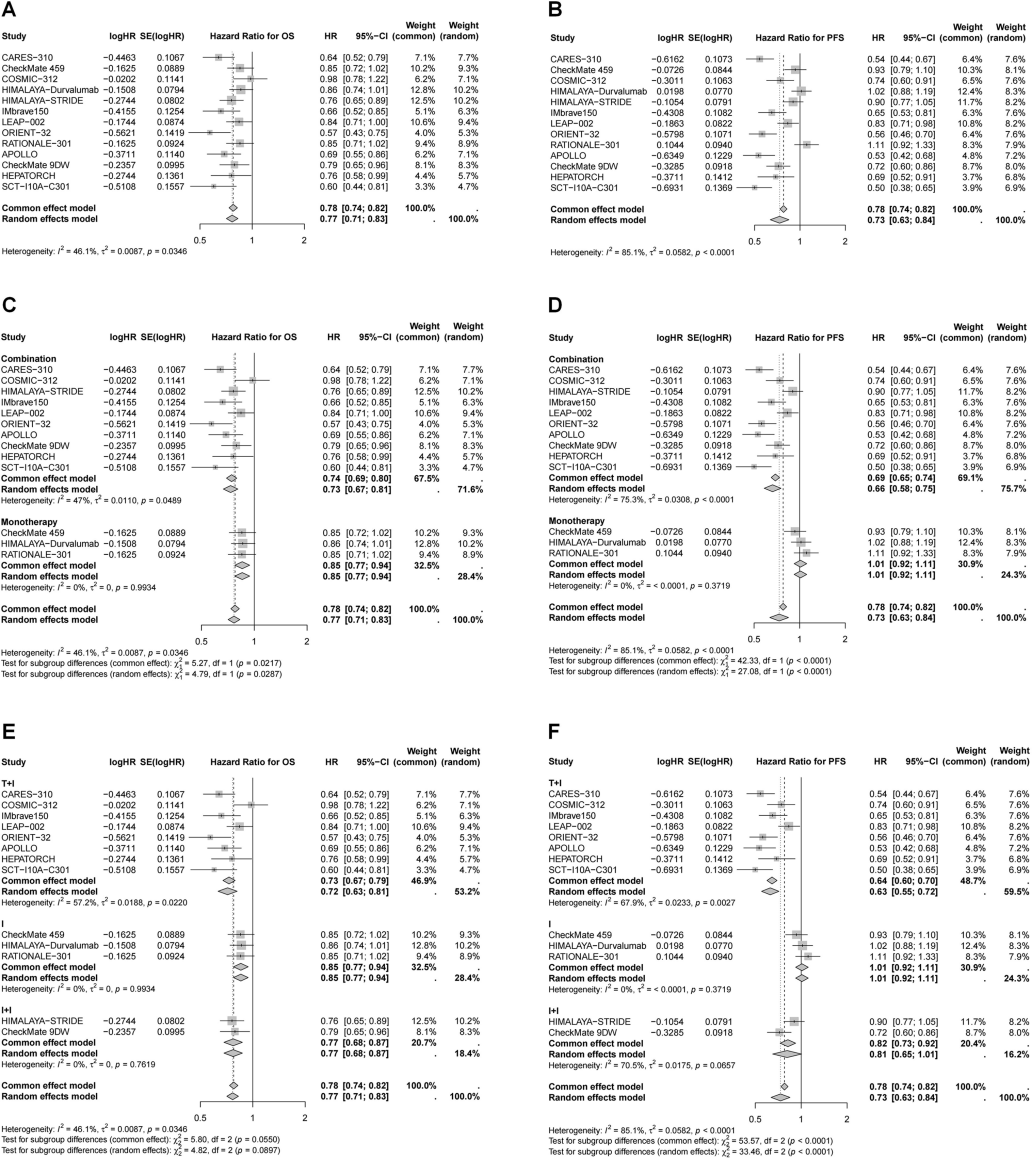
Forest plots of pooled HR for OS and PFS. **(A)** HR for OS in whole population; **(B)** HR for PFS in whole population; **(C)** HR for OS stratified by different treatment strategies; **(D)** HR for PFS stratified by different treatment strategies; **(E)** HR for OS stratified by different combination therapies; **(F)** HR for PFS stratified by different combination therapies.

All included studies reported OS and PFS outcomes (**Figure 2**). In general, immunotherapy significantly improved OS compared to the control group (HR = 0.77, 95% CI 0.71–0.83), with combination therapy providing the most pronounced benefit (27% reduction in mortality risk *vs*. 15% for monotherapy, *P* < 0.05). Although not statistically significant, ICI plus targeted therapy showed the greatest reduction in mortality risk (27%), followed by dual ICIs (23%). Immunotherapy also significantly improved PFS (HR = 0.73, 95% CI 0.63–0.84), particularly in patients receiving ICI plus targeted therapy, which led to a 37% reduction in disease progression risk. In contrast, the benefits of dual ICIs and monotherapy were more limited.

**Figure 3:**
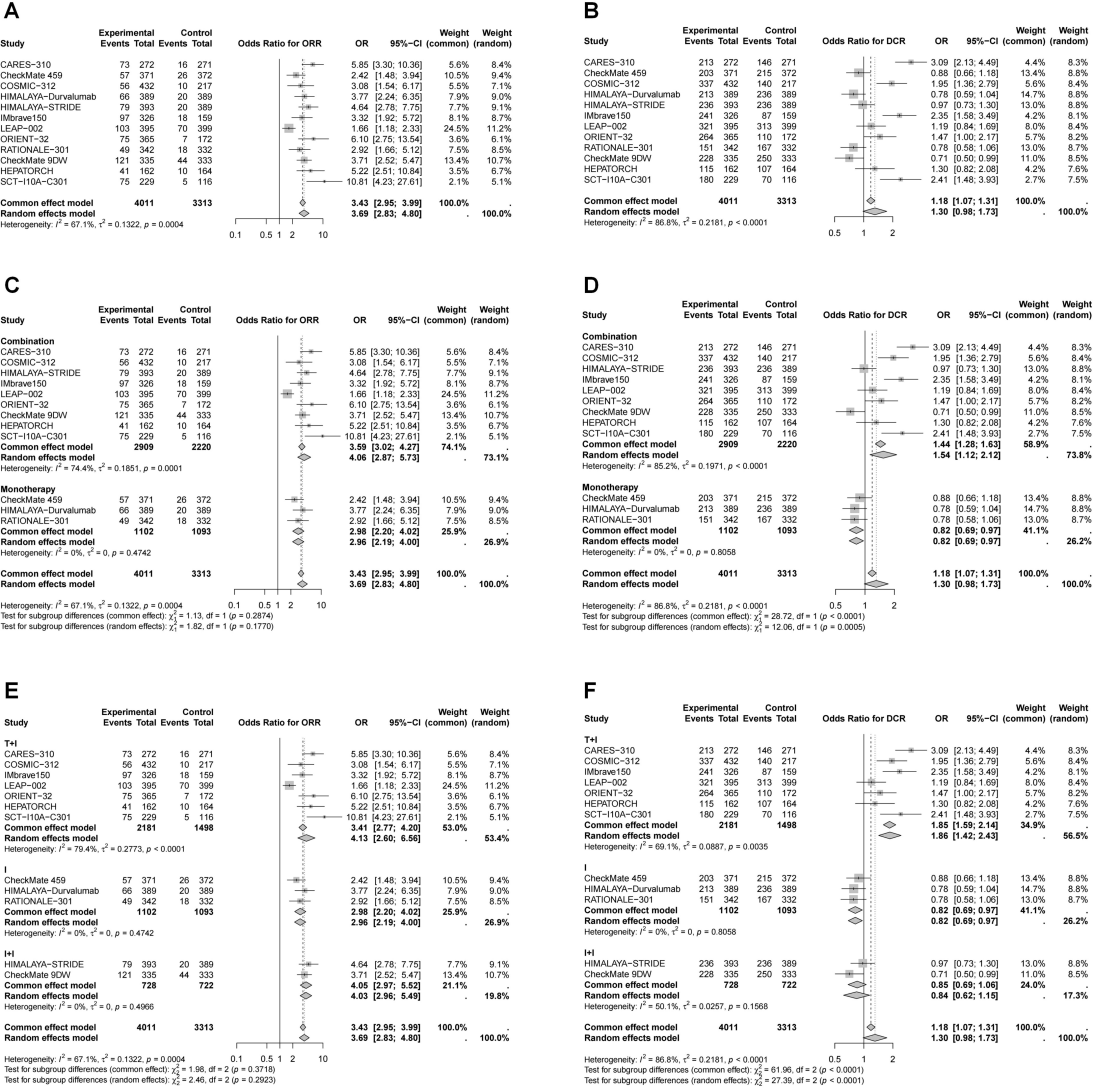
Forest plots of pooled OR for ORR and DCR by RECIST v1.1. **(A)** OR for ORR in whole population; **(B)** OR for DCR in whole population; **(C)** OR for ORR stratified by different treatment strategies; **(D)** OR for DCR stratified by different treatment strategies; **(E)** OR for ORR stratified by different combination therapies; **(F)** OR for DCR stratified by different combination therapies.

Except for one study, all trials reported ORR and DCR assessed by Response Evaluation Criteria in Solid Tumors (RECIST) v1.1 (**Figure 3**). The pooled ORR analysis demonstrated a significant advantage of immunotherapy over the control (OR = 3.69, 95% CI 2.83–4.80). The highest ORs were observed for combination therapy (ICI plus targeted therapy: OR = 4.13, 95% CI 2.60–6.56; dual ICIs: OR = 4.05, 95% CI 2.97–5.52). However, immunotherapy did not show a significant advantage in DCR compared to the control group (OR = 1.30, 95% CI 0.98–1.73). Subgroup analysis indicated that monotherapy and dual ICIs therapy had limited effects on DCR, while ICI plus targeted therapy significantly improved DCR (OR = 1.86, 95% CI 1.42–2.43). Pooled ORs for ORR and DCR assessed by modified RECIST (mRECIST) are presented in **Supplementary Figure 2**.

The incidence of adverse events is shown in **Supplementary Figure 3**. Compared to TKIs, immunotherapy was associated with a lower risk of TRAEs (OR = 0.46, 95% CI 0.29–0.74), with monotherapy demonstrating the lowest incidence (OR = 0.18, 95% CI 0.14–0.23), followed by dual ICIs therapy (OR = 0.53, 95% CI 0.40–0.71). However, the risk of TRAEs in ICI plus targeted therapy was not significantly different from the control group (OR = 0.74, 95% CI 0.45–1.22). Regarding serious TRAEs (grade 3-5), no significant difference was found between immunotherapy and the control group (OR = 0.86, 95% CI 0.55–1.35). Nonetheless, ICI monotherapy exhibited a lower rate of severe TRAEs (OR = 0.26, 95% CI 0.22–0.32).

### Efficacy of immunotherapy in different subgroup based on age and gender

**Figure 4:**
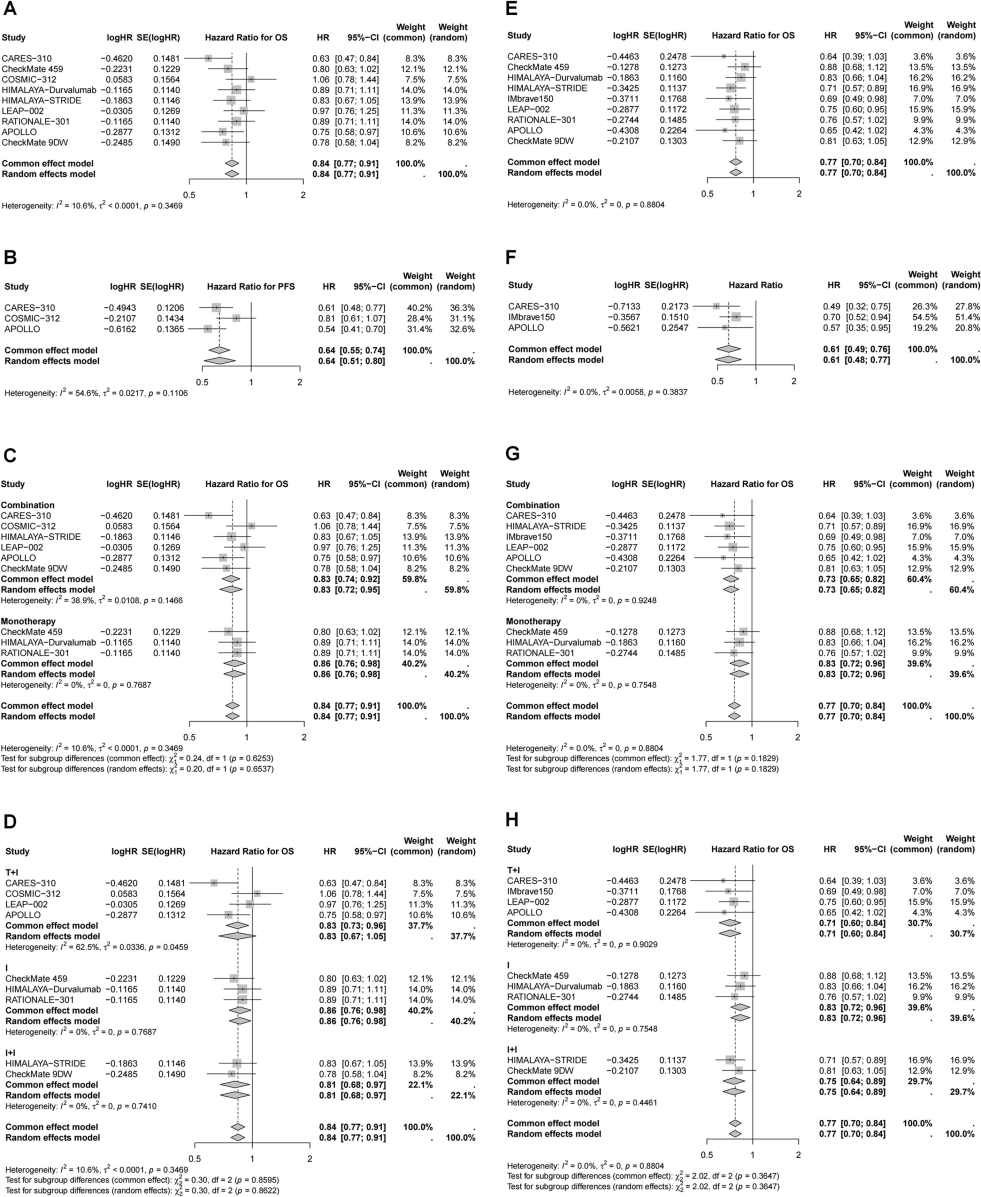
Forest plots of pooled HR for OS and PFS in different age. **(A)** OS for age < 65 years; **(B)** PFS for age < 65 years; **(C)** OS for age < 65 years stratified by different treatment strategies; **(D)** OS for age < 65 years stratified by different combination therapies; **(E)** OS for age ≥ 65 years; **(F)** PFS for age ≥ 65 years; **(G)** OS for age ≥ 65 years stratified by different treatment strategies; **(H)** OS for age ≥ 65 years stratified by different combination therapies.

**Figure 5:**
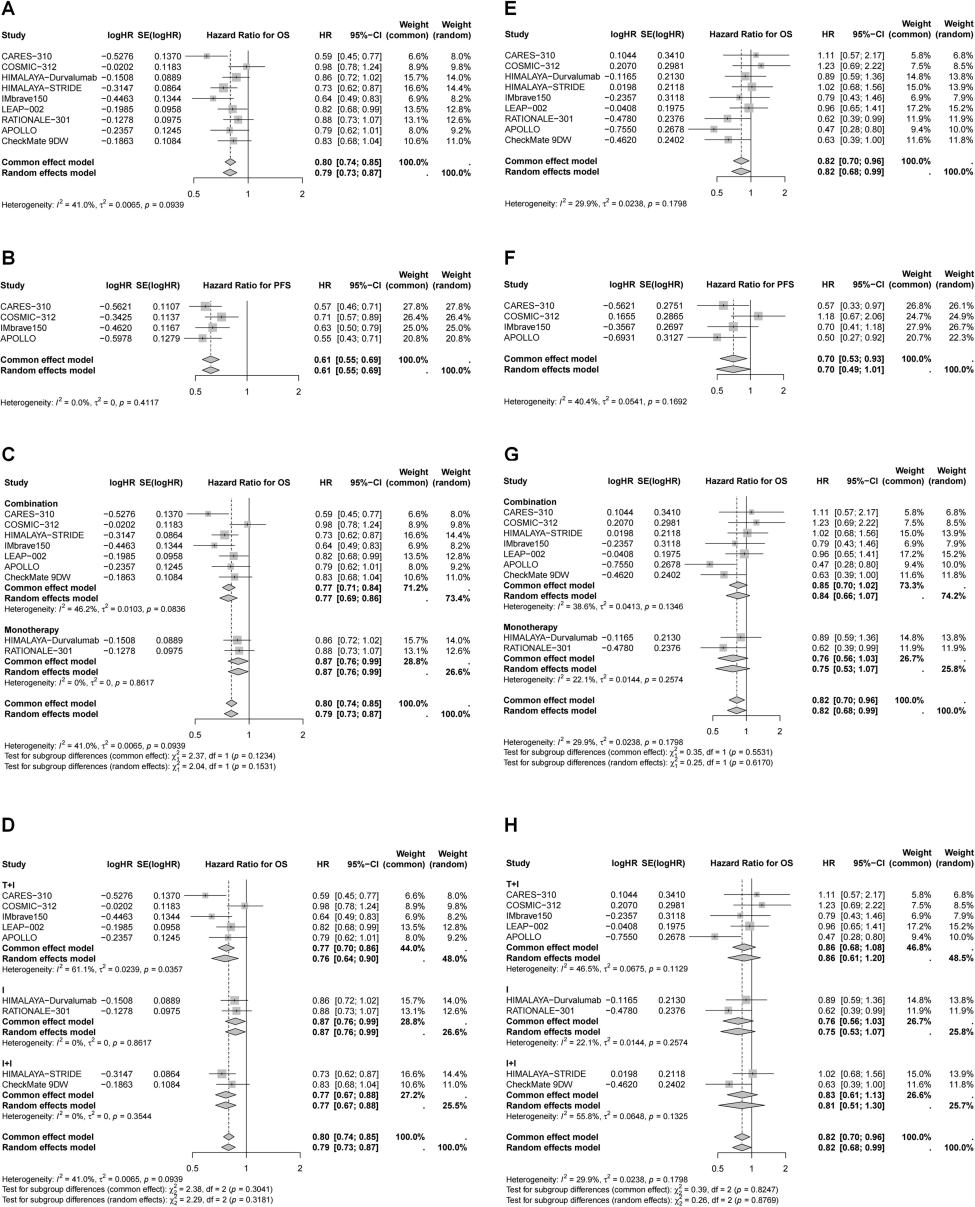
Forest plots of pooled HR for OS and PFS in different gender. **(A)** OS for male; **(B)** PFS for male; **(C)** OS for male stratified by different treatment strategies; **(D)** OS for male stratified by different combination therapies; **(E)** OS for female; **(F)** PFS for female; **(F)** OS for female stratified by different treatment strategies; **(G)** OS for female stratified by different combination therapies.

Subgroup analyses were performed according to age and sex (**Tables 2**, **3**, **Figures 4**, **5**). Patients aged ≥65 years and male patients derived greater OS and PFS benefits from immunotherapy (**Table 2**, **Figures 4**, **5**). Specifically, in patients aged ≥65 years, the greatest reduction in mortality risk was observed with ICI plus targeted therapy (29%), followed by dual ICIs (25%) and ICI monotherapy (17%). In contrast, among younger patients, dual ICIs conferred the most favorable survival outcomes (HR for OS = 0.81, 95% CI 0.68–0.97), whereas ICI plus targeted therapy failed to provide significant benefit (HR for OS = 0.83, 95% CI 0.67–1.05). For male patients, ICI plus targeted therapy yielded the largest reduction in mortality risk (24%), followed by dual ICIs (23%) and monotherapy (13%). Notably, none of the immunotherapy regimens—monotherapy, dual ICIs, or ICI plus targeted therapy—significantly improved OS in female patients.

**Table 2:** Pooled HR for OS and PFS in different subgroups.

	HR for OS (95% CI)	HR for PFS (95% CI)
All patients	0.77 (0.71, 0.83)	0.73 (0.63, 0.84)
Age, years		
<65	0.84 (0.77, 0.91)	0.64 (0.51, 0.80)
≥65	0.77 (0.70, 0.84)	0.61 (0.49, 0.76)
Sex		
Male	0.79 (0.73, 0.87)	0.61 (0.55, 0.69)
Female	0.82 (0.70, 0.96)	0.70 (0.53, 0.93)
Geographic region		
Asia	0.77 (0.70, 0.84)	0.55 (0.46, 0.65)
Non-Asia	0.86 (0.78, 0.93)	0.80 (0.67, 0.95)
Etiology		
HBV	0.73 (0.67, 0.80)	0.54 (0.48, 0.61)
HCV	0.78 (0.64, 0.94)	0.70 (0.53, 0.92)
Uninfected	0.87 (0.78, 0.96)	0.82 (0.65, 1.03)
ECOG PS		
0	0.83 (0.76, 0.90)	0.64 (0.56, 0.73)
1	0.73 (0.67, 0.80)	0.59 (0.51, 0.68)
MVI		
Yes	0.70 (0.62, 0.79)	0.54 (0.46, 0.64)
No	0.83 (0.77, 0.89)	0.65 (0.54, 0.78)
EHS		
Yes	0.73 (0.66, 0.82)	0.58 (0.51, 0.65)
No	0.85 (0.77, 0.94)	0.70 (0.59, 0.82)
MVI/EHS		
Yes	0.75 (0.70, 0.80)	0.57 (0.51, 0.64)
No	0.91 (0.80, 1.04)	0.75 (0.61, 0.91)
BCLC stage		
B	0.87 (0.74, 1.03)	0.75 (0.60, 0.94)
C	0.77 (0.72, 0.82)	0.58 (0.53, 0.65)
AFP, ng/mL		
<400	0.83 (0.74, 0.94)	0.62 (0.49, 0.77)
≥400	0.72 (0.66, 0.79)	0.57 (0.43, 0.75)

AFP, α-Fetoprotein; BCLC, Barcelona Clinic Liver Cancer; CI, confidence interval; ECOG, Eastern Cooperative Oncology Group; EHS, extra hepatic spread; HBV, hepatitis B virus; HCV, hepatitis C virus; HR, hazard ratio; MVI, macrovascular invasion; OS, overall survival; PFS, progression-free survival; PS, performance status.

**Table 3:** Pooled HR for OS (95% CI) in different subgroups stratified by different therapies.

OS	Combination	T+I	I+I	I
All	0.73 (0.67, 0.81)	0.72 (0.63, 0.81)	0.77 (0.68, 0.87)	0.85 (0.77, 0.94)
Age, years				
<65	0.83 (0.74, 0.92)	0.83 (0.67, 1.05)	0.81 (0.68, 0.97)	0.86 (0.76, 0.98)
≥65	0.73 (0.65, 0.82)	0.71 (0.60, 0.84)	0.75 (0.64, 0.89)	0.83 (0.72, 0.96)
Sex				
Male	0.77 (0.69, 0.86)	0.76 (0.64, 0.90)	0.77 (0.67, 0.88)	0.87 (0.76, 0.99)
Female	0.85 (0.70, 1.02)	0.86 (0.68, 1.08)	0.81 (0.51, 1.30)	0.76 (0.56, 1.03)
Geographic region				
Asia	0.72 (0.63, 0.81)	0.71 (0.61, 0.83)	0.72 (0.59, 0.88)	0.84 (0.73, 0.97)
Non-Asia	0.83 (0.70, 1.00)	0.83 (0.64, 1.07)	0.82 (0.67, 1.01)	0.86 (0.75, 0.99)
Etiology				
HBV	0.69 (0.62, 0.76)	0.67 (0.60, 0.75)	0.75 (0.60, 0.93)	0.84 (0.73, 0.98)
HCV	0.75 (0.56, 1.01)	0.69 (0.43, 1.12)	0.82 (0.64, 1.06)	0.82 (0.66, 1.02)
Uninfected	0.87 (0.77, 0.99)	0.96 (0.80, 1.16)	0.79 (0.65, 0.95)	0.86 (0.73, 1.00)
ECOG PS				
0	0.81 (0.73, 0.89)	0.80 (0.65, 0.99)	0.79 (0.67, 0.92)	0.88 (0.75, 1.03)
1	0.70 (0.63, 0.78)	0.68 (0.60, 0.77)	0.75 (0.61, 0.91)	0.83 (0.69, 0.98)
MVI				
Yes	0.66 (0.58, 0.75)	0.65 (0.55, 0.76)	0.69 (0.54, 0.87)	0.87 (0.68, 1.11)
No	0.82 (0.72, 0.92)	0.80 (0.67, 0.96)	0.83 (0.72, 0.95)	0.85 (0.74, 0.97)
EHS				
Yes	0.70 (0.62, 0.79)	0.68 (0.58, 0.80)	0.75 (0.63, 0.88)	0.85 (0.73, 0.99)
No	0.86 (0.77, 0.97)	0.91 (0.78, 1.06)	0.79 (0.66, 0.95)	0.82 (0.68, 0.98)
MVI/EHS				
Yes	0.71 (0.65, 0.78)	0.69 (0.63, 0.77)	0.76 (0.66, 0.89)	0.81 (0.73, 0.91)
No	0.90 (0.77, 1.06)	0.96 (0.80, 1.15)	0.74 (0.52, 1.04)	0.94 (0.65, 1.36)
BCLC stage				
B	0.85 (0.69, 1.04)	0.86 (0.62, 1.20)	0.76 (0.58, 1.01)	0.93 (0.66, 1.30)
C	0.74 (0.68, 0.80)	0.72 (0.65, 0.79)	0.78 (0.68, 0.89)	0.84 (0.75, 0.93)
AFP, ng/ml				
<400	0.81 (0.68, 0.96)	0.79 (0.61, 1.02)	0.84 (0.71, 1.00)	0.88 (0.77, 1.01)
≥400	0.69 (0.60, 0.80)	0.70 (0.58, 0.84)	0.66 (0.52, 0.83)	0.75 (0.63, 0.89)

AFP, α-Fetoprotein; BCLC, Barcelona Clinic Liver Cancer; CI, confidence interval; ECOG, Eastern Cooperative Oncology Group; EHS, extra hepatic spread; HBV, hepatitis B virus; HCV, hepatitis C virus; HR, hazard ratio; I+I, dual ICIs; I, ICI monotherapy; MVI, macrovascular invasion; OS, overall survival; PS, performance status; T+I, ICI plus targeted therapy.

### Efficacy of immunotherapy in different subgroup based on region, etiology, and tumor burden

Further subgroup analyses were conducted based on geographic region, etiology, performance status (PS), presence of macrovascular invasion (MVI) and/or extra hepatic spread (EHS), Barcelona Clinic Liver Cancer (BCLC) stage, and α-Fetoprotein (AFP) levels (**Tables 2**, **3**, **Supplementary Figures 4–12**).

Overall, patients who were Asian, had a higher tumor burden, or presented with AFP ≥400 ng/mL demonstrated greater OS and PFS benefits from immunotherapy (**Table 2**). With respect to etiology, HBV-positive patients achieved superior OS, PFS, and ORR compared with HCV-infected patients and those without viral hepatitis (OS: 0.73 *vs*. 0.78 *vs*. 0.87; PFS: 0.54 *vs*. 0.70 *vs*. 0.82; ORR: 3.93 *vs*. 3.08 *vs*. 3.02) (**Table 2**, **Supplementary Figure 5**).

Moreover, Asian patients, HBV-infected patients, those with ECOG PS = 1, MVI and/or EHS, and BCLC stage C derived the greatest OS improvements from ICI plus targeted therapy compared with dual ICIs and monotherapy (**Table 3**). In contrast, for patients without viral infection, with ECOG PS = 0, without EHS, and with AFP ≥400 ng/mL, dual ICI therapy was more beneficial, particularly in patients with AFP ≥400 ng/mL (HR = 0.66, 95% CI 0.52-0.83) (**Table 3**). Interestingly, non-Asian patients, HCV-infected patients, and those with BCLC stage B or AFP <400 ng/mL appeared to derive only limited benefit from immunotherapy, regardless of whether they receive ICI plus targeted therapy, dual ICIs, or monotherapy (**Table 3**).

### Publication bias detection

Begg’s and Egger’s tests were conducted to assess publication bias. The *P*-values for Begg’s test were 0.0441, 0.0124, 0.0467, and 0.0236 for OS, PFS, ORR, and DCR, respectively. Similarly, Egger’s test yielded *P*-values of 0.0237, 0.0023, 0.0058, and 0.0107 for these respective outcomes, indicating potential publication bias. Additionally, asymmetry in the funnel plots suggested the presence of publication bias (**Supplementary Figure 13**).

The Baujat plot (**Supplementary Figure 14**) identified key studies contributing to heterogeneity: ORIENT-32 had the most substantial impact on heterogeneity for OS measurement, while COSMIC-312 influenced the overall results for OS measurement. For PFS measurement, RATIONALE-301 contributed most to heterogeneity, while HIMALAYA-Durvalumab had the greatest effect on overall results. LEAP-002 had the most significant influence on heterogeneity and overall results for ORR measurement, while CARES-310 impacted heterogeneity and pooled results for DCR measurement. Sensitivity analysis demonstrated slight variations in pooled HRs for OS and PFS, as well as ORs for DCR when individual studies were removed (**Supplementary Figure 15**). Notably, removing LEAP-002 reduced I² to 30%, indicating its considerable influence on overall effect estimates of ORR.

## Discussion

This meta-analysis provides a comprehensive evaluation of the efficacy and safety of immunotherapeutic regimens in unresectable HCC. Our findings reinforce the transformative impact of ICIs in HCC management, especially when used in combination with targeted therapies, and highlight important subgroup differences that may guide personalized treatment decisions.

Our analysis demonstrated that immunotherapy significantly improved OS and PFS compared to traditional TKIs. Generally, HCC is intrinsically an inflammation-driven malignancy, with viral infections—particularly hepatitis B and C—playing a predominant etiological role (39–41). Persistent infection induces prolonged hepatic inflammation, which establishes a distinct immune landscape characterized by suppression of immune activity. This immunological adaptation not only minimizes autoimmune-mediated liver injury but also compromises antitumor immunity, thereby enabling tumor cells to evade immune surveillance and facilitating HCC development.

Recent reviews emphasize that the therapeutic potential of ICIs in HCC can be maximized through rational combination strategies that modulate both tumor-intrinsic and microenvironmental resistance mechanisms (42, 43). HCC exhibits pronounced heterogeneity in immune-cell infiltration and the composition of the tumor microenvironment (TME). This diversity—characterized by T-cell exhaustion, enrichment of immunosuppressive cell populations such as regulatory T cells (Tregs), myeloid-derived suppressor cells, and M2-like macrophages, and extensive fibrotic remodeling—gives rise to immune-inflamed, immune-excluded, and immune-desert phenotypes (42, 43). Such TME variability contributes to wide differences in responses to immunotherapy and underscores the need for combination strategies capable of enhancing immune activation and improving clinical outcomes. Currently, the ORR of ICI monotherapy remains below 20%, and only a subset of patients experience durable benefit. Combination regimens—including ICIs with TKIs, ICIs with chemotherapy, and dual ICIs approaches—are rapidly emerging as central therapeutic options for HCC. Understanding the specific immune barriers targeted by each combination is essential for optimizing patient outcomes and enabling precision immunotherapy (42). The rationale for integrating TKIs with ICIs in HCC arises from their complementary mechanisms within the TME. Notably, in our analysis the benefit was most pronounced in patients receiving ICI plus targeted therapy, which achieved a 27% reduction in mortality risk and a 37% reduction in disease progression risk. HCC is a highly vascularized malignancy typified by aberrant angiogenesis, which fosters an immunosuppressive microenvironment that not only promotes tumor progression but also impedes immunotherapeutic efficacy (42). TKIs exert regulatory effects on the tumor microenvironment by simultaneously targeting multiple signaling pathways, including vascular endothelial growth factor receptors (VEGFR) and fibroblast growth factor receptors (FGFR) (44, 45). The VEGF signaling pathway plays a pivotal role within the tumor microenvironment. Its inhibition can remodel the aberrant tumor vasculature, thereby facilitating the infiltration of effector immune cells, enhancing the functional activity of effector T cells, and reducing the abundance of immunosuppressive cell populations (46, 47). Combination therapy with targeted agents and ICI reshapes the tumor microenvironment and reinforces T cell-mediated immunity, thereby shifting the tumor milieu from an immunosuppressive to an immune-activated state, ultimately improving the therapeutic efficacy of ICIs (48, 49). Several TKI–ICI combinations have been approved as first-line therapies and consistently demonstrate survival advantages across diverse patient populations. Although ICI plus targeted therapy showed the largest numerical reduction in mortality (27%), the difference was not statistically significant, likely due to between-study heterogeneity, variable follow-up durations, and limited statistical power across trials. As longer-term data mature, clearer distinctions among combination regimens may emerge. Furthermore, subgroups such as patients with low tumor burden may derive differential benefit from dual ICIs therapy. Dual ICIs therapy targeting PD-1/PD-L1 and CTLA-4 is supported by the distinct yet synergistic roles of these pathways in T-cell regulation. PD-1/PD-L1 primarily restricts effector T-cell activity within the TME, whereas CTLA-4 governs early T-cell priming in lymphoid tissues. Concurrent blockade enhances both the initiation and execution of antitumor immunity. This strategy is especially effective in HCCs with an immune-inflamed phenotype—tumors that already contain infiltrating and activated T cells—whereas immune-desert tumors may require additional priming interventions. The therapeutic landscape of dual-checkpoint inhibition is expanding to incorporate emerging targets such as LAG-3 and TIGIT (42). A broader spectrum of combination strategies—such as pairing ICIs with chemotherapy, integrating ICIs with interventional therapies, combining ICIs with ablative techniques, or employing multifunctional materials to enhance drug delivery and targeting—may also offer promising therapeutic avenues for HCC (42, 43). Collectively, personalized combination immunotherapy—guided by etiologic background, immune status, and molecular context—represents a critical next step toward improving long-term outcomes in unresectable HCC. Emerging technologies, including single-cell RNA sequencing, spatial transcriptomics, and multi-omics integration, will facilitate the identification of predictive biomarkers and support rational treatment matching (42).

The immune system undergoes significant alterations with aging and the process of tumor (1, 50, 51). Different clinical characteristics may affect immunotherapy efficacy for cancer, including age (52), sex (52, 53), ECOG (54), and so on. The study demonstrated that patients who were of Asian ethnicity, older age, male, HBV-infected, had an ECOG PS of 1, presented with MVI and/or EHS, advanced stage, or elevated AFP levels (≥400 ng/mL) exhibited greater improvements in OS and PFS following immunotherapy. Our subgroup analyses provide further insights into the differential benefits of immunotherapy. The enhanced survival outcomes observed in Asian populations, HBV-associated HCC, and male patients confirm previous findings (10–13). The apparent benefit among patients of Asian ethnicity likely reflects, at least in part, differences in underlying disease etiology and tumor immunobiology. In many Asian populations HBV infection predominates as the major cause of HCC (55); chronic viral antigen exposure can shape an inflamed tumor microenvironment with upregulated immune checkpoint expression (e.g., PD-1/PD-L1), potentially rendering tumors more susceptible to checkpoint blockade (56). In addition, population-specific host genetics (e.g., HLA alleles and immune-regulatory polymorphisms), environmental exposures, and microbiome composition may modulate systemic and intrahepatic immunity and thereby influence responsiveness to immunotherapy (57). The enhanced efficacy of immunotherapy in older patients may be linked to age-associated immune remodeling. Aging is accompanied by profound alterations in both the composition and functionality of the immune system, a process known as immunosenescence (58). Immunosenescence exerts complex and sometimes paradoxical effects on tumor immunity. Aging reshapes both systemic and hepatic immunity by reducing cytotoxic T-cell infiltration and increasing Tregs, thereby fostering an immunosuppressive TME. Although immunosenescence weakens immune surveillance, it also results in the accumulation of PD-1–high exhausted T cells, which may respond more effectively to checkpoint blockade. Moreover, HBV or HCV infection accelerates immune aging through persistent antigenic stimulation and inflammatory stress. HBV upregulates inhibitory receptors such as PD-1, TIM-3, and LAG-3, while HCV induces telomere shortening and chronic inflammation—conditions that sensitize tumors to ICI-mediated immune reactivation. Aging-related gut microbiome dysbiosis and elevated tumor mutational burden (TMB) may further enhance tumor immunogenicity and treatment response (58, 59). Collectively, these factors suggest that immunosenescence does not uniformly suppress antitumor immunity but reprograms it, creating a state in which checkpoint inhibitors can partially restore immune competence and improve therapeutic outcomes in elderly HCC patients. Sex-based differences in immunotherapy response are influenced by sex chromosomes, sex hormones, and immune-related gene expression (60, 61). Females generally exhibit stronger innate and adaptive immune responses, which confer better protection against infections but predispose them to autoimmune diseases such as rheumatoid arthritis. In contrast, males often show weaker innate immunity and greater T-cell exhaustion, leading to poorer outcomes in viral infections and certain cancers. Interestingly, PD-1 blockade therapy appears more effective in males, possibly due to androgen-driven expansion of progenitor exhausted T cells that sustain antitumor immunity. ECOG PS = 1, MVI and/or EHS, and advanced BCLC stage are markers of higher tumor burden and aggressive biology; such patients may harbor more immunogenic tumors (higher antigen load or inflammatory infiltrates) and thus exhibit a larger absolute benefit from treatments that augment antitumor immunity. We emphasize caution in interpretation because PS is also a composite clinical measure influenced by comorbidities and performance status ascertainment. Tumors with extensive vascular invasion or metastatic spread may present an increased load of tumor-associated antigens and greater neoantigen heterogeneity, which can enhance T-cell recognition after checkpoint inhibition. Furthermore, locoregional vascular abnormalities and hypoxia in such tumors may be alleviated by combination approaches (e.g., TKI plus ICIs), thereby these populations may represent patients with more aggressive tumor biology or a higher tumor burden, for whom the addition of targeted therapy may help modulate the tumor microenvironment and enhance the efficacy of immunotherapy (62, 63). Intriguingly, in subgroups defined by younger age (<65 years), absence of viral hepatitis, and high AFP levels, dual ICIs therapy appeared to offer a greater survival advantage. One plausible explanation is that these patients might have tumors with distinct immunogenic profiles, making them more responsive to the robust immune activation elicited by dual checkpoint inhibition. Younger patients may benefit more from dual ICIs because they exhibit a more active and diverse immune repertoire. They retain higher frequencies of functional effector and memory T cells and greater T cell receptor diversity, enabling stronger reinvigoration under combined PD-1/CTLA-4 blockade. Moreover, immunosuppressive populations such as regulatory CD4^+^ T cells and myeloid-derived suppressor cells are relatively lower in younger individuals, creating a less inhibitory tumor microenvironment (64–66). These characteristics constitute a distinct immunogenic profile that favors enhanced response to dual checkpoint inhibition. Non-viral HCC, particularly non-alcoholic steatohepatitis (NASH)- or alcohol-associated disease, is characterized by chronic metabolic inflammation and fibrosis that profoundly remodel the immune microenvironment. NASH drives the accumulation of IgA^+^ immunosuppressive plasmocytes, a major source of PD-L1 and IL-10, which directly induce CD8^+^ T-cell exhaustion and impair antitumor surveillance (67). In parallel, dysfunctional NK cells, Th17 cells, and PD-1^+^ CD8^+^ T cells further establish an immunosuppressed milieu, whereas alcohol-related liver disease promotes intrahepatic expansion of tumor-promoting myeloid-derived suppressor cells (68–70). Together, these features reduce tumor immunogenicity and may explain the limited benefit of immunotherapy in non-viral HCC. In particular, high AFP levels, which are often associated with aggressive tumor behavior, may indicate a subset of patients whose tumors are more immunogenic and thus more likely to respond to the enhanced immune modulation provided by dual ICIs (71). Moreover, AFP itself exerts immunosuppressive effects by impairing dendritic-cell antigen presentation, inhibiting NK-cell cytotoxicity, and suppressing CD4^+^ and CD8^+^ T-cell proliferation (72–74). These findings underscore the heterogeneity of HCC and suggest that tailored treatment strategies based on patient and disease characteristics are warranted.

Immune-related adverse events represent a key consideration in selecting between different immunotherapy regimens. Serious adverse events are associated with the use of ICI, especially in combination (75–77). Overall, immunotherapy was associated with a lower risk of TRAEs compared with TKIs, with ICI monotherapy showing the lowest incidence. Analysis of grade ≥3 TRAEs further illustrated distinct safety patterns. ICI monotherapy showed the lowest incidence (12.9–22.3%; OR = 0.26), consistent with its narrower immune activation profile. Dual ICIs demonstrated moderately higher rates (28.1–44.9%; OR = 0.83), reflecting broader checkpoint blockade–driven immune toxicity. In contrast, ICI plus targeted therapy exhibited the highest rates (34.5–80.9%; OR = 1.36), probably attributable to VEGF/TKI-related and immune-mediated adverse effects. Although steroid-requiring AEs were not reported, these results underscore the need to balance efficacy with tolerability, particularly when selecting regimens for clinically vulnerable subgroups.

We also found that the subgroup—non-Asian patients, females, those with HCV infection, BCLC stage B, or baseline AFP < 400 ng/mL—showed limited benefit from immunotherapy, regardless of whether they receive ICI plus targeted therapy, dual ICIs, or monotherapy. This pattern likely reflects differences in tumor biology, immune microenvironment, and patient characteristics that influence ICI efficacy. In non-Asian populations, HCC is primarily caused by HCV infection or NAFLD, differing from the HBV-related HCC more common in Asia (78). These etiological distinctions result in diverse tumor immune microenvironments (TIME), which may influence the effectiveness of immunotherapy. HBV-related HCC tends to be more immunogenic, which may explain better responses to immune checkpoint inhibitors (79). These findings highlight the need to consider underlying etiology and immune context when selecting immunotherapy approaches for HCC. In females, estrogen signaling has been implicated in modulating immune checkpoints and cytokine profiles, potentially leading to a more suppressive TIME (80–82). Further prospective studies stratifying by sex are required to clarify these observations. Patients with BCLC-B HCC frequently receive locoregional therapies and exhibit more heterogeneous tumor burden and vascular patterns compared with advanced-stage disease. This heterogeneity can affect ICI delivery and the composition of tumor-infiltrating lymphocytes. Moreover, vascular invasion and macrovascular thrombi—more common in advanced stages—have been associated with greater benefit from ICIs. Thus, BCLC B patients without these high-risk features may have tumors less reliant on checkpoint-mediated immune escape, translating into lower immunotherapy responsiveness. AFP reflects aggressive tumor biology and burden. A previous study has shown that antigen processing and interferon-γ response–related genes are highly expressed in AFP-positive HCC tumor cells. Additionally, AFP-positive HCC is characterized by widespread immune dysregulation, including T cell exhaustion (83). These features suggest that ICIs may restore antitumor immunity by reversing immune cell exhaustion through blockade of inhibitory signals within the tumor microenvironment. This implies that the immunostimulatory effects of ICIs might be attenuated in AFP-low HCC.

The findings of this meta-analysis have important implications for clinical practice. They support the preferential use of combination immunotherapy strategies in the first-line setting for unresectable HCC, particularly in patient subgroups with high-risk features. Moreover, our results highlight the need for further research to delineate the optimal therapeutic regimen for specific populations and to better understand the mechanisms underlying differential treatment responses. Future studies should focus on well-designed, large-scale randomized controlled trials that incorporate comprehensive biomarker analyses and long-term follow-up to further validate these findings.

Despite these promising findings, our study has several limitations. First, the majority of the included trials were open-label, which introduces a risk of performance bias. Additionally, the inclusion of conference abstracts, with their limited methodological details, may have contributed to uncertainties in data interpretation. Publication bias was also detected via Begg’s and Egger’s tests, indicating that the pooled estimates should be interpreted with caution. Finally, although our analysis included a greater number of RCTs than previous meta-analyses, heterogeneity in patient populations, treatment regimens, and outcome assessments remains a challenge (84). These factors underscore the need for future large-scale, well-designed trials to validate our findings and further explore the differential benefits observed across various subgroups.

## Conclusion

In summary, our analysis not only reinforces the survival benefits of immunotherapy in HCC but also clarifies the nuanced differences between various immunotherapeutic strategies. The findings provide critical insights that may inform clinical decision-making and ultimately improve patient outcomes in this challenging malignancy.

## Data Availability

The original contributions presented in the study are included in the article/**Supplementary Material**. Further inquiries can be directed to the corresponding authors.
