# Supplementary material for: Survival benefits of different immunotherapies for hepatocellular carcinoma: a meta-analysis highlighting age, gender, etiology, and tumor burden
Source: Front Immunol. 2025 Dec 10;16:1713151. doi: 10.3389/fimmu.2025.1713151 (PMC12728079; doi:10.3389/fimmu.2025.1713151)
Supplement: Supplementary file 1 [file DataSheet1.docx]

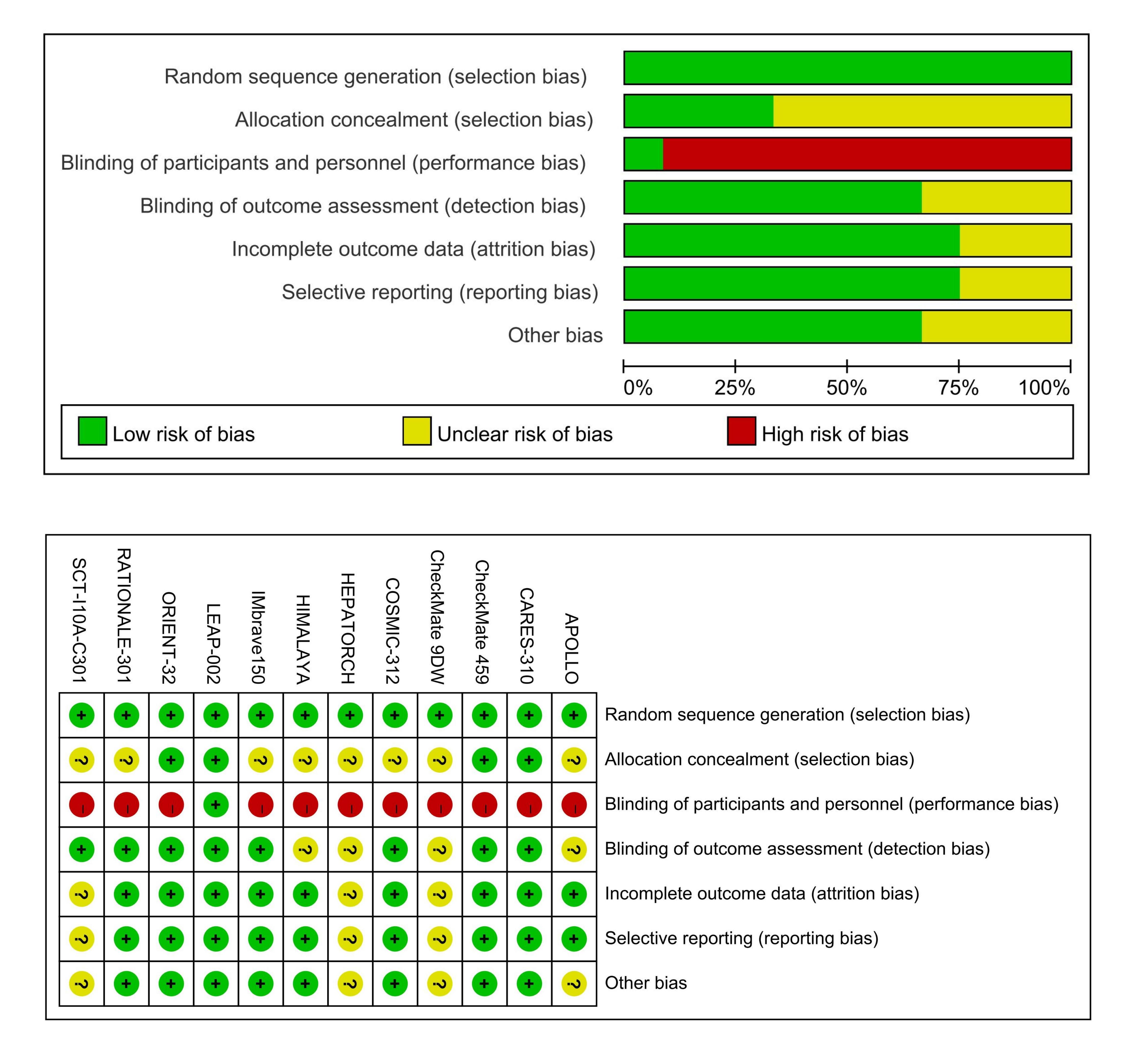


**Supplementary Figure 1.** Risk of bias graph.


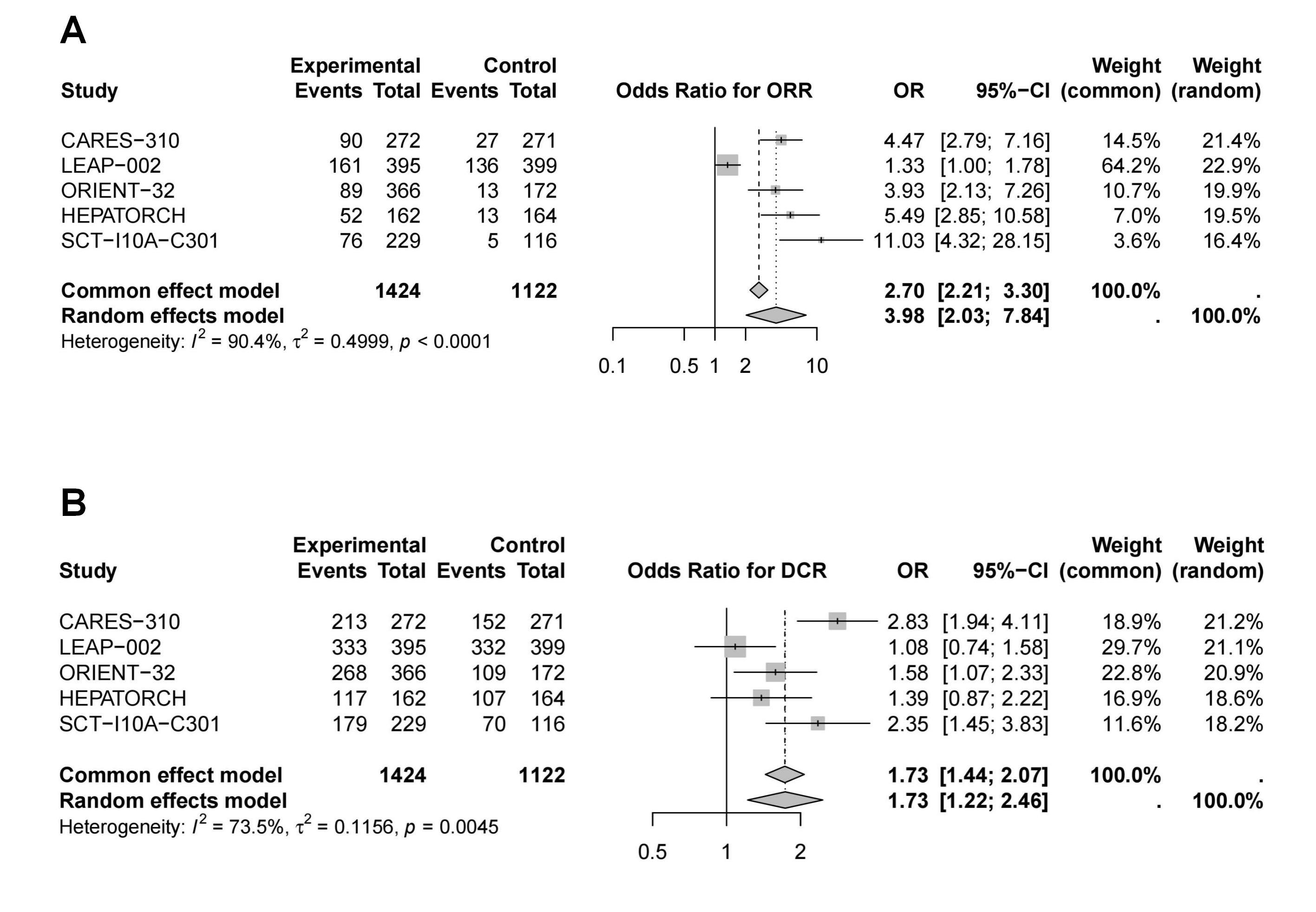


**Supplementary Figure 2.** Forest plots of pooled OR for ORR and DCR by mRECIST. A) OR for ORR in all studies; B) OR for DCR in all studies.


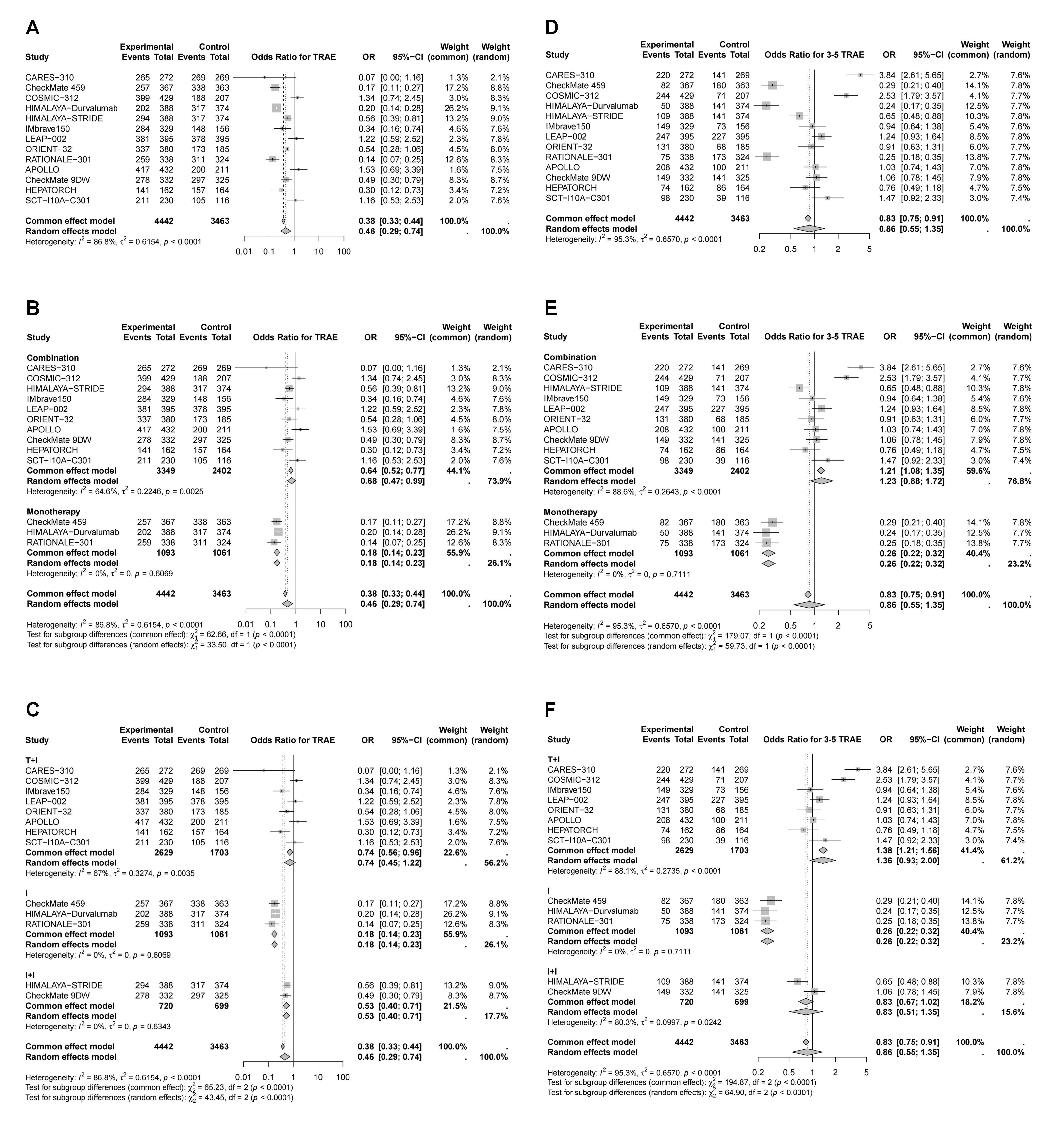


**Supplementary Figure 3.** Forest plots of pooled OR for any grade and grade 3–5 TRAEs. A) OR for any grade TRAEs in whole population; B) OR for any grade TRAEs stratified by different treatment strategies; C) OR for any grade TRAEs stratified by different combination therapies; D) OR for grade 3–5 TRAEs in whole population; E) OR for grade 3–5 TRAEs stratified by different treatment strategies; F) OR for grade 3–5 TRAEs stratified by different combination therapies.


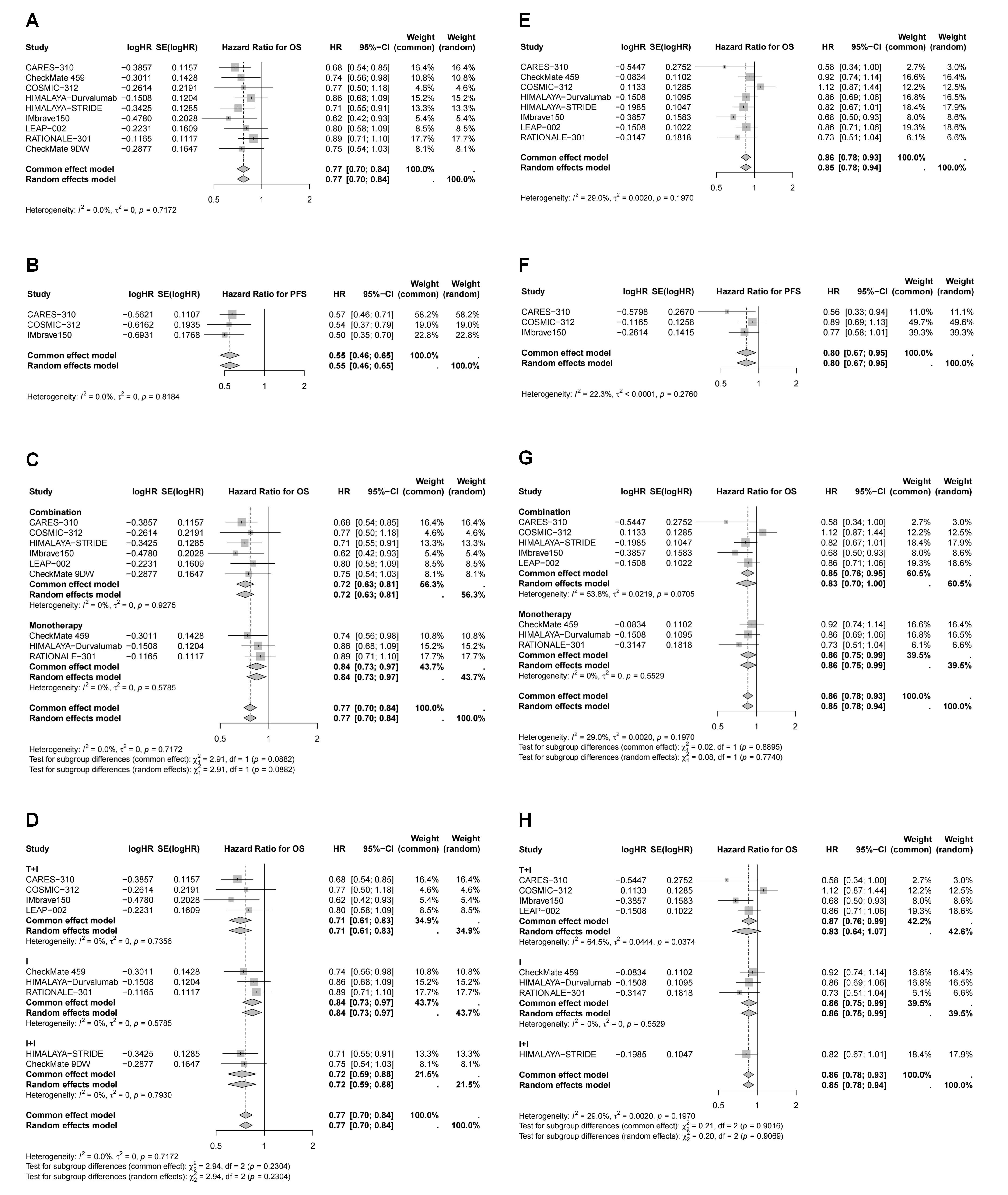


**Supplementary Figure 4.** Forest plots of pooled HR for OS and PFS in different geographic region. A) OS for Asia; B) PFS for Asia; C) OS for Asia stratified by different treatment strategies; D) OS for Asia stratified by different combination therapies; E) OS for non-Asia; F) PFS for non-Asia; G) OS for non-Asia stratified by different treatment strategies; H) OS for non-Asia stratified by different combination therapies.


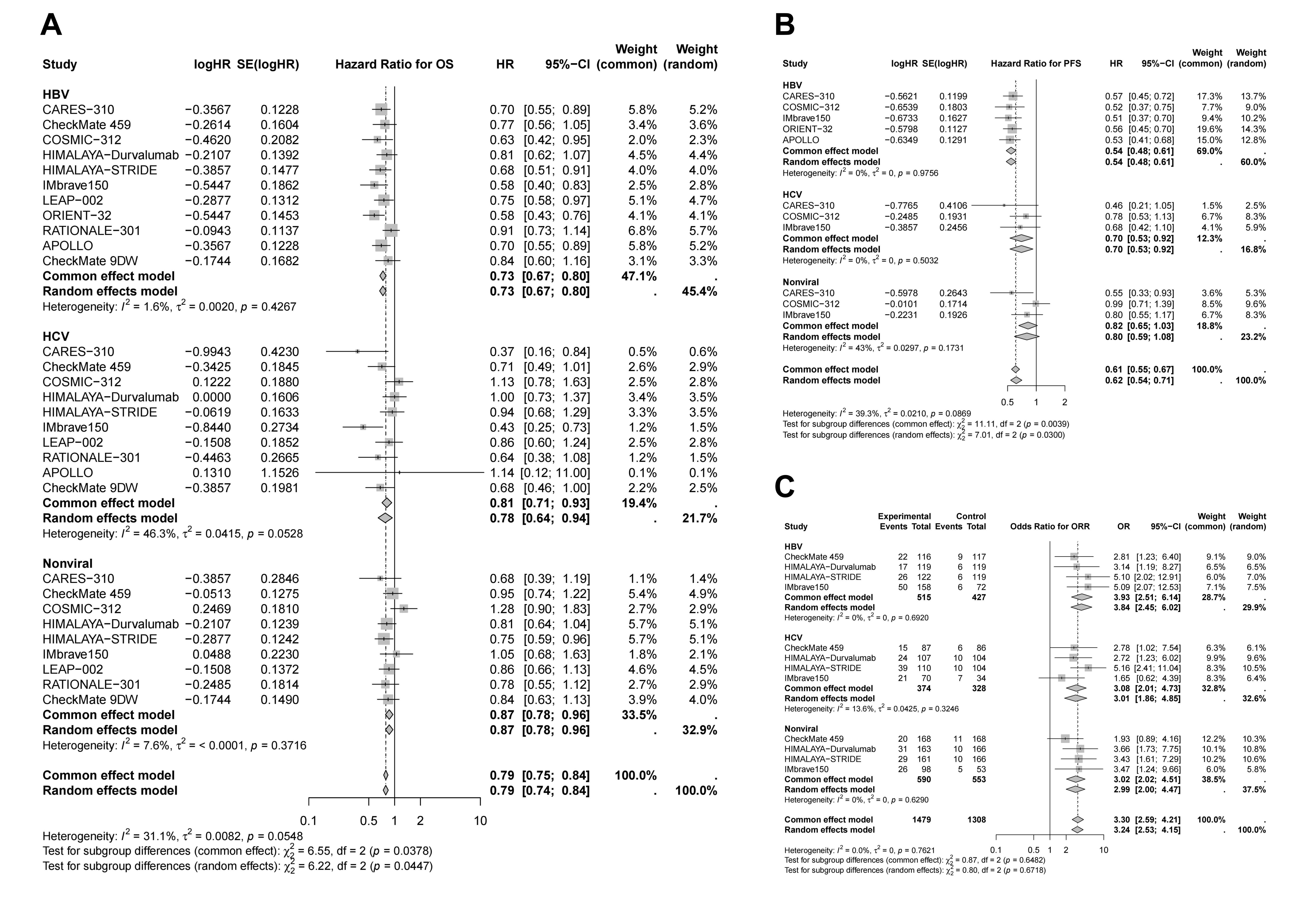


**Supplementary Figure 5.** Forest plots of pooled HR for OS and PFS and OR for ORR in different etiologies. A) HR for OS; B) HR for PFS; C) OR for ORR by RECIST v1.1.


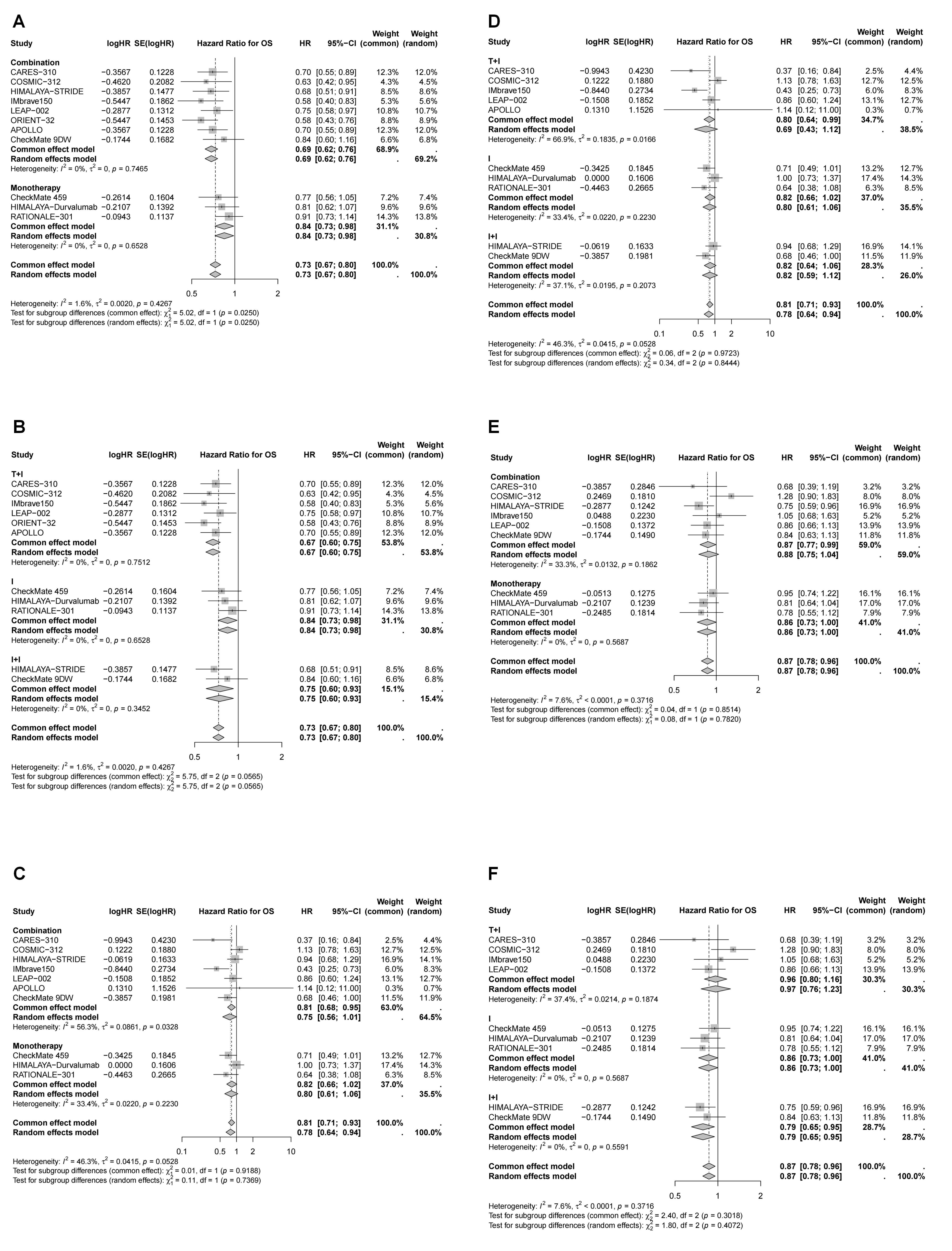


**Supplementary Figure 6.** Forest plots of pooled HR for OS in different etiologies stratified by different therapies. A) OS for HBV-HCC stratified by different treatment strategies; B) OS for HBV-HCC stratified by different combination therapies; C) OS for HCV-HCC stratified by different treatment strategies; D) OS for HCV-HCC stratified by different combination therapies; E) OS for nonviral-HCC stratified by different treatment strategies; F) OS for nonviral-HCC stratified by different combination therapies.


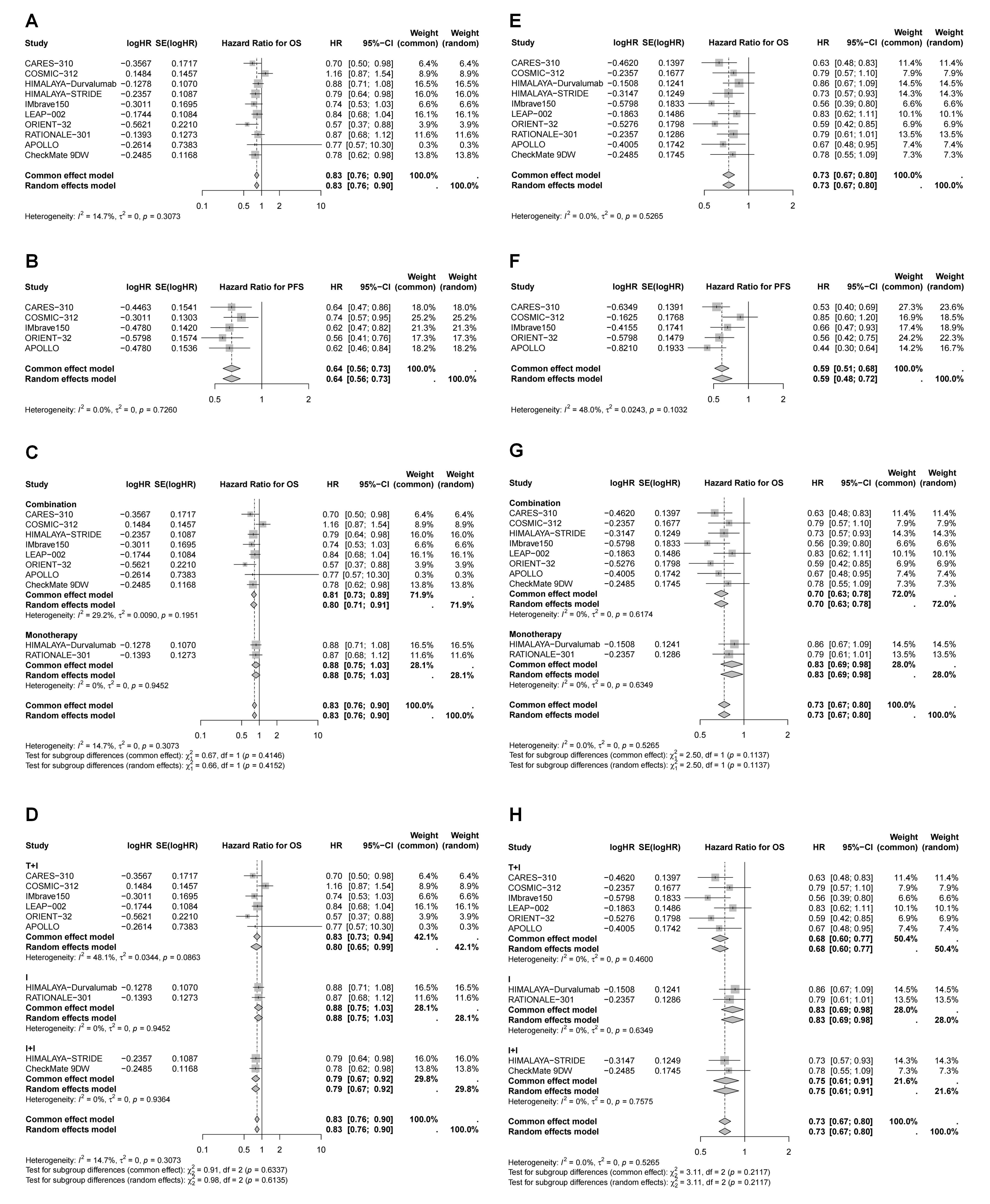


**Supplementary Figure 7.** Forest plots of pooled HR for OS and PFS in different performance status. A) OS for ECOG 0; B) PFS for ECOG 0; C) OS for ECOG 0 stratified by different treatment strategies; D) OS for ECOG 0 stratified by different combination therapies; E) OS for ECOG 1; F) PFS for ECOG 1; G) OS for ECOG 1stratified by different treatment strategies; H) OS for ECOG 1 stratified by different combination therapies.


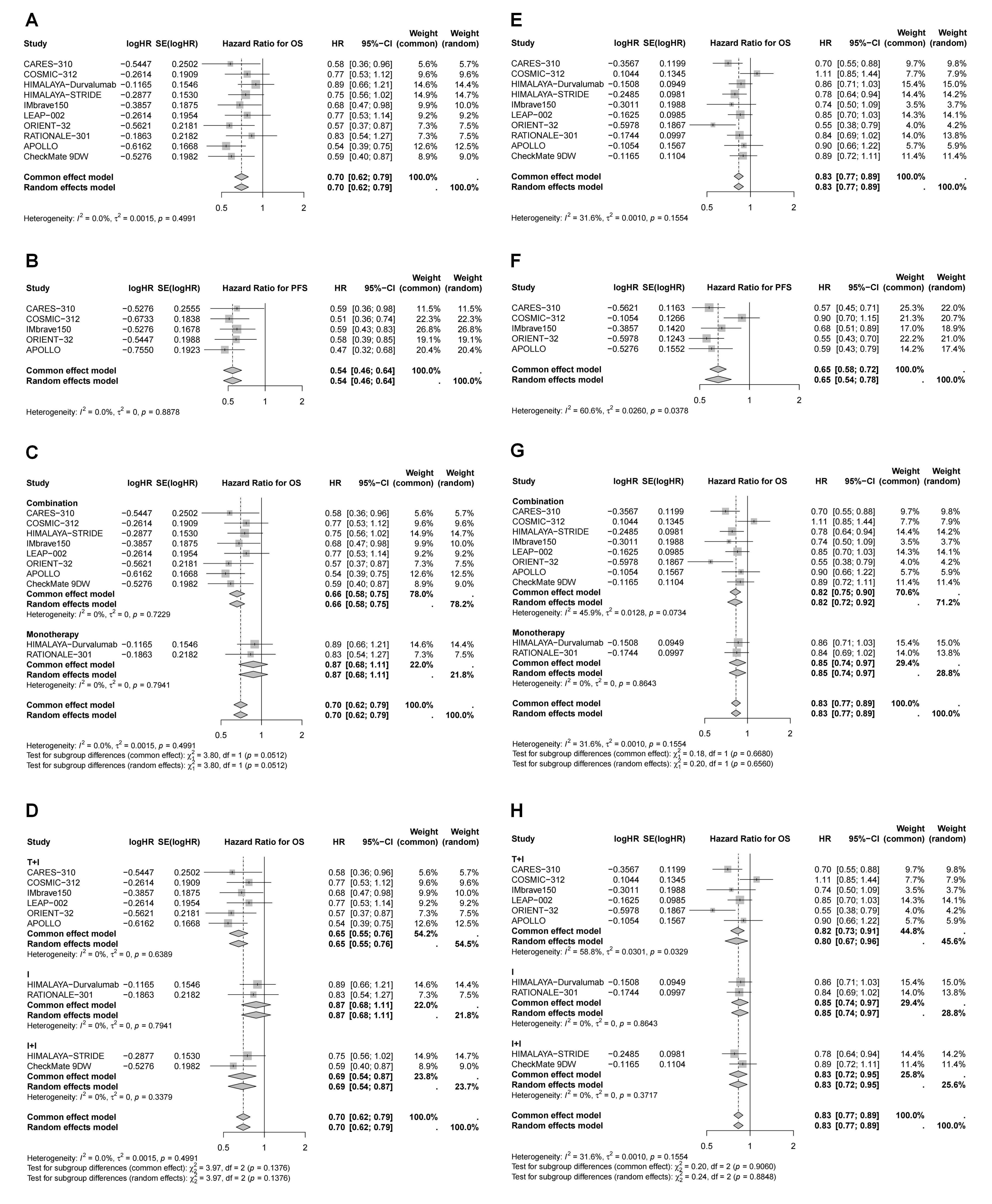


**Supplementary Figure 8.** Forest plots of pooled HR for OS and PFS in the presence/absence of MVI. A) OS for HCC with MVI; B) PFS for HCC with MVI; C) OS for HCC with MVI stratified by different treatment strategies; D) OS for HCC with MVI stratified by different combination therapies; E) OS for HCC without MVI; F) PFS for HCC without MVI; G) OS for HCC without MVI stratified by different treatment strategies; H) OS for HCC without MVI stratified by different combination therapies.


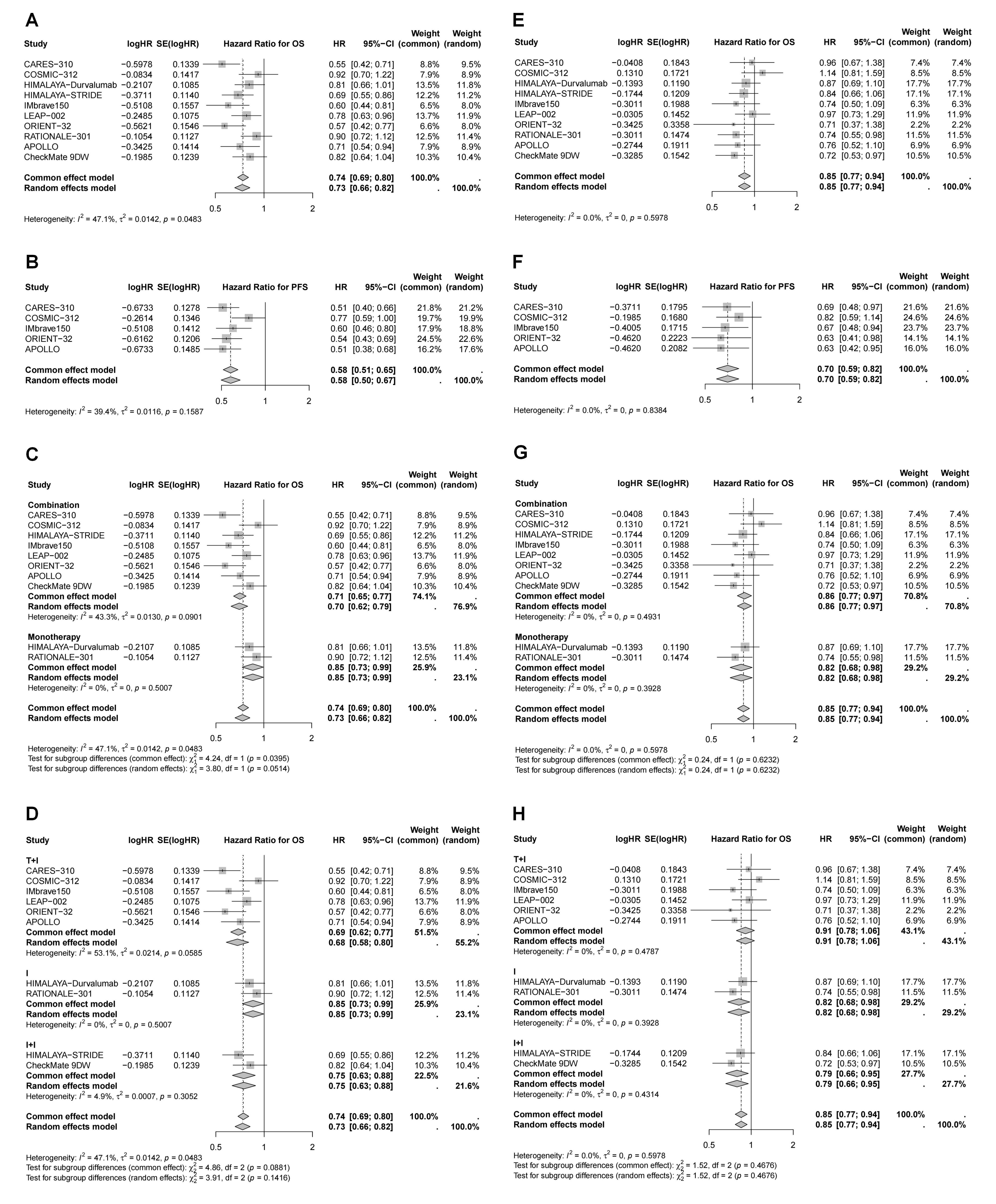


**Supplementary Figure 9.** Forest plots of pooled HR for OS and PFS stratified by the presence/absence of EHS. A) OS for HCC with EHS; B) PFS for HCC with EHS; C) OS for HCC with EHS stratified by different treatment strategies; D) OS for HCC with EHS stratified by different combination therapies; E) OS for HCC without EHS; F) PFS HCC without EHS; G) OS for HCC without EHS stratified by different treatment strategies; H) OS for HCC without EHS stratified by different combination therapies.


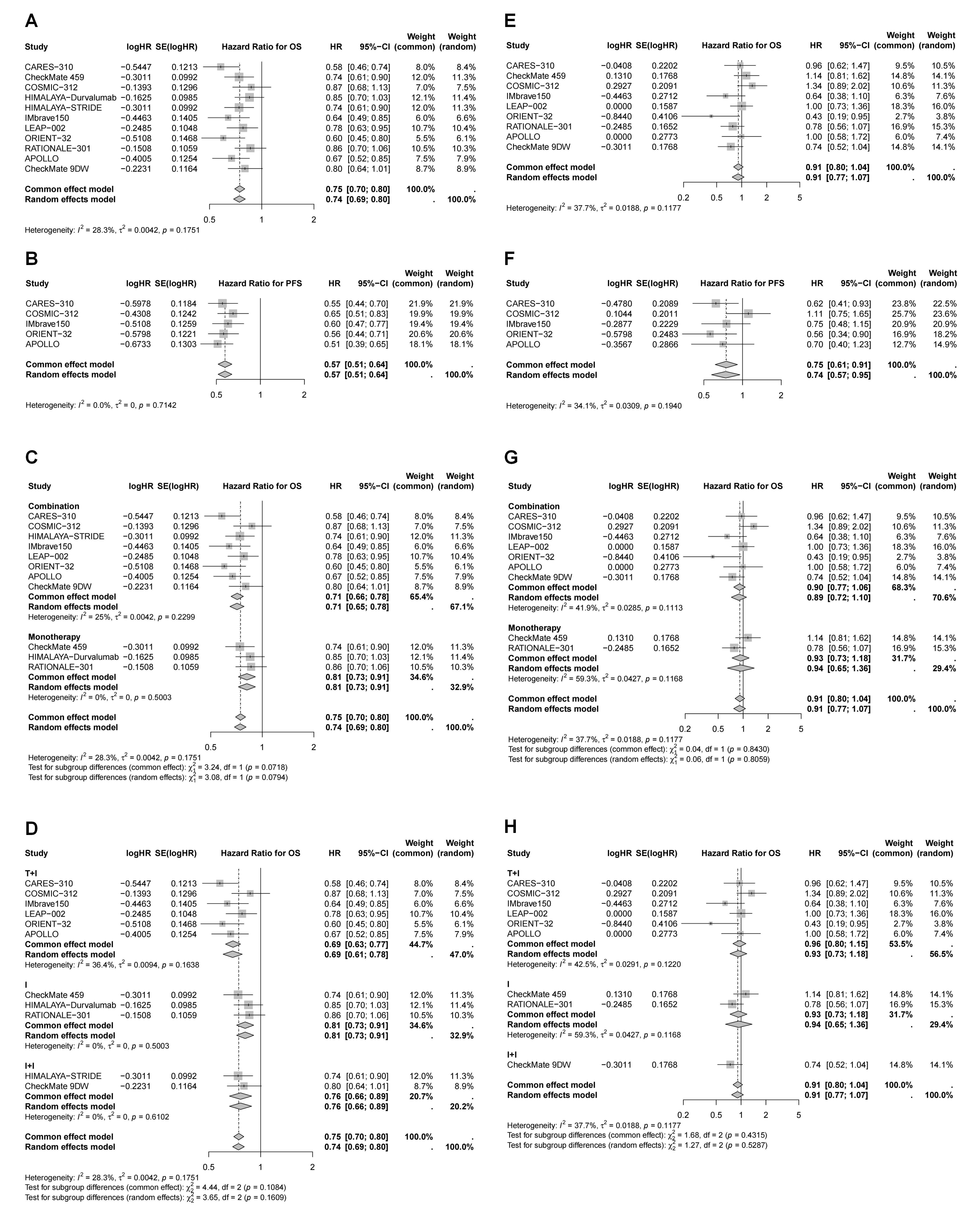


**Supplementary Figure 10.** Forest plots of pooled HR for OS and PFS stratified by the presence/absence of MVI/EHS. A) OS for HCC with MVI/EHS; B) PFS for HCC with MVI/EHS; C) OS for HCC with MVI/EHS stratified by different treatment strategies; D) OS for HCC with MVI/EHS stratified by different combination therapies; E) OS for HCC without MVI/EHS; F) PFS HCC without MVI/EHS; G) OS for HCC without MVI/EHS stratified by different treatment strategies; H) OS for HCC without MVI/EHS stratified by different combination therapies.


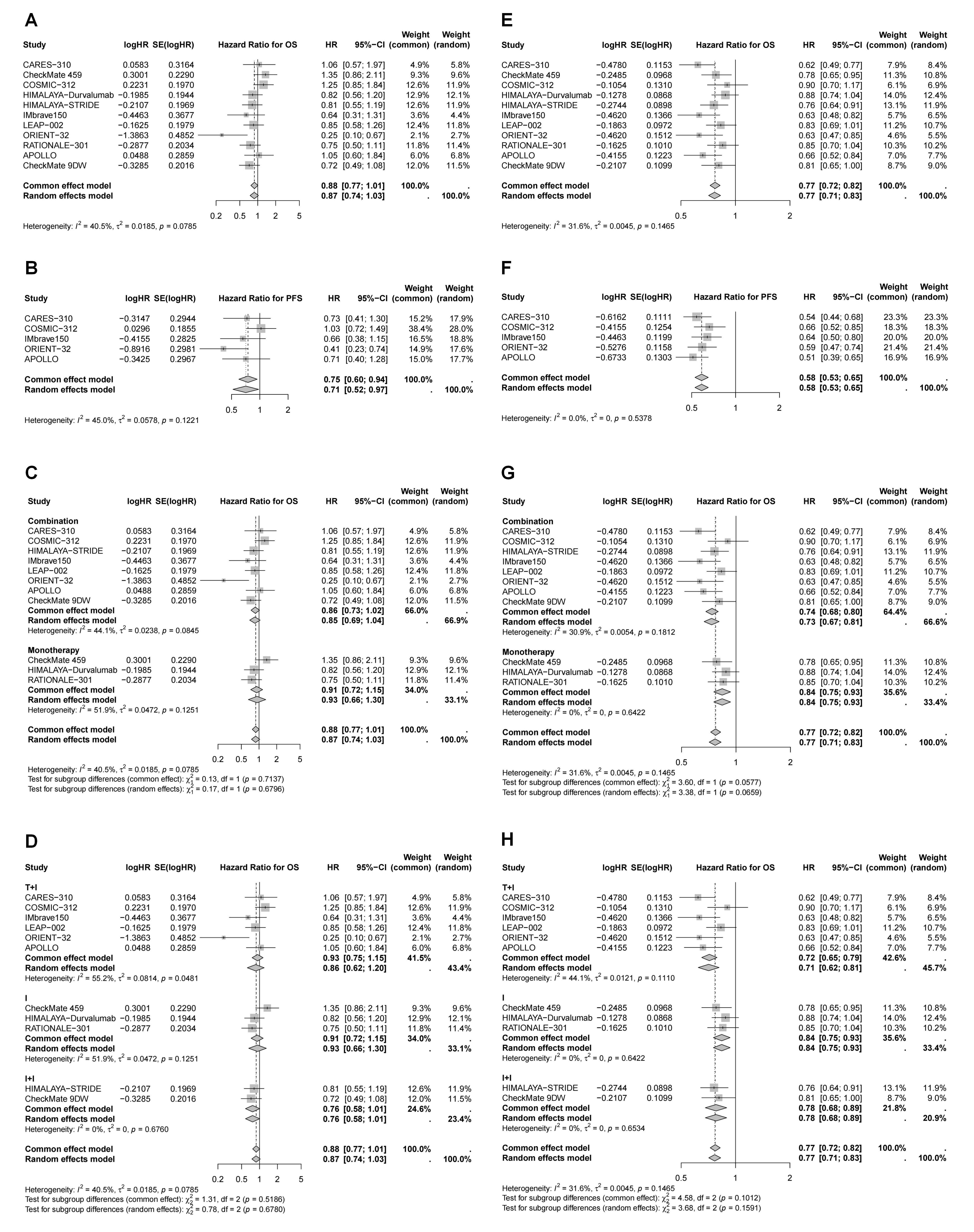


**Supplementary Figure 11.** Forest plots of pooled HR for OS and PFS stratified by BCLC stage. A) OS for stage B; B) PFS for stage B; C) OS for stage B stratified by different treatment strategies; D) OS for stage B stratified by different combination therapies; E) OS for stage C; F) PFS for stage C; G) OS for stage C stratified by different treatment strategies; H) OS for stage C stratified by different combination therapies.


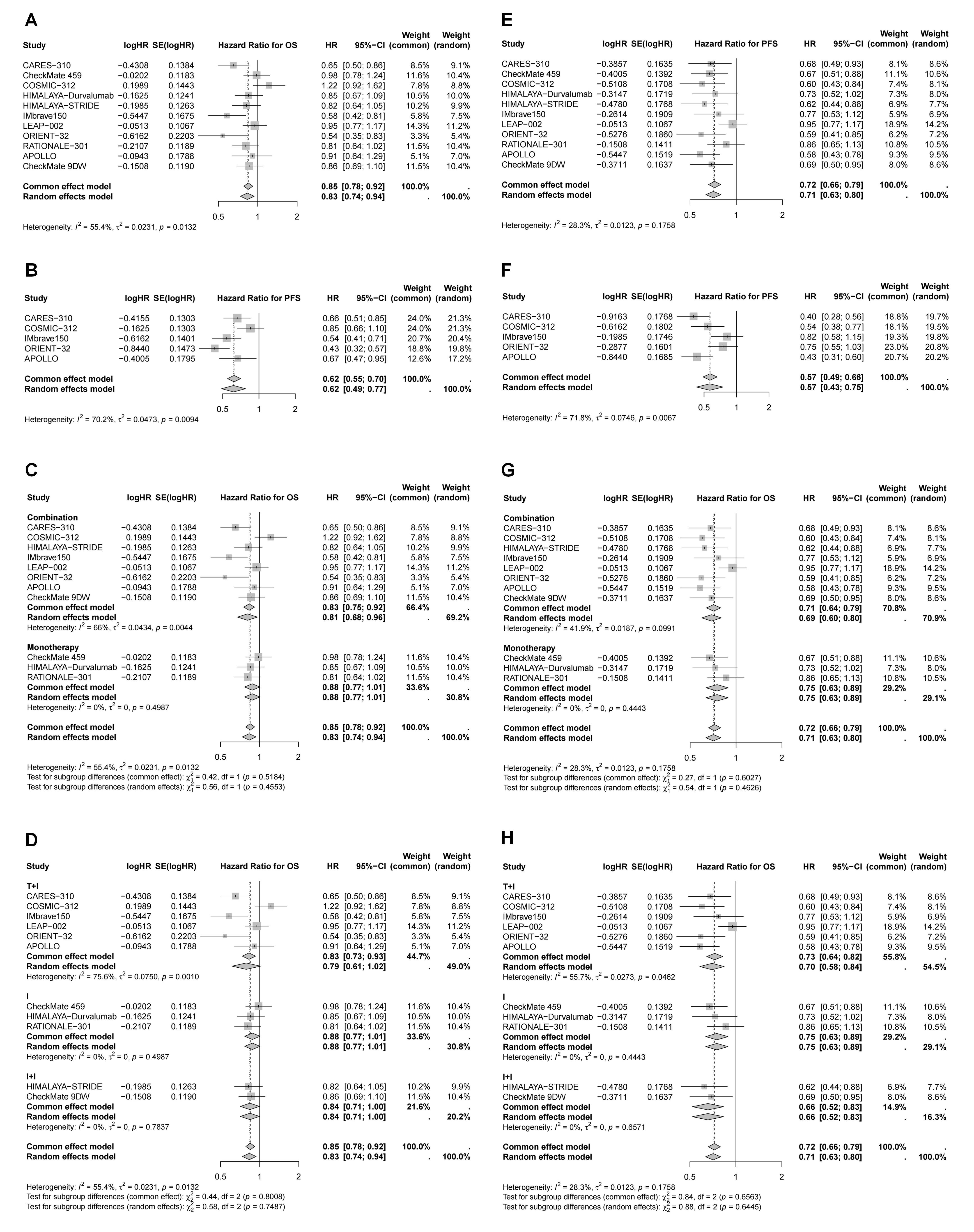


**Supplementary Figure 12.** Forest plots of pooled HR for OS and PFS stratified by AFP levels. A) OS for AFP < 400ng/mL; B) PFS for AFP < 400ng/mL; C) OS for AFP < 400ng/mL stratified by different treatment strategies; D) OS for AFP < 400ng/mL stratified by different combination therapies; E) OS for AFP ≥ 400ng/mL; F) PFS for AFP ≥ 400ng/mL; G) OS for AFP ≥ 400ng/mL stratified by different treatment strategies; H) OS for AFP ≥ 400ng/mL stratified by different combination therapies.





**Supplementary Figure 13.** Funnel plot for detecting publication bias. A) Funnel plot for OS; B) Funnel plot for PFS; C) Funnel plot for ORR; D) Funnel plot for DCR.


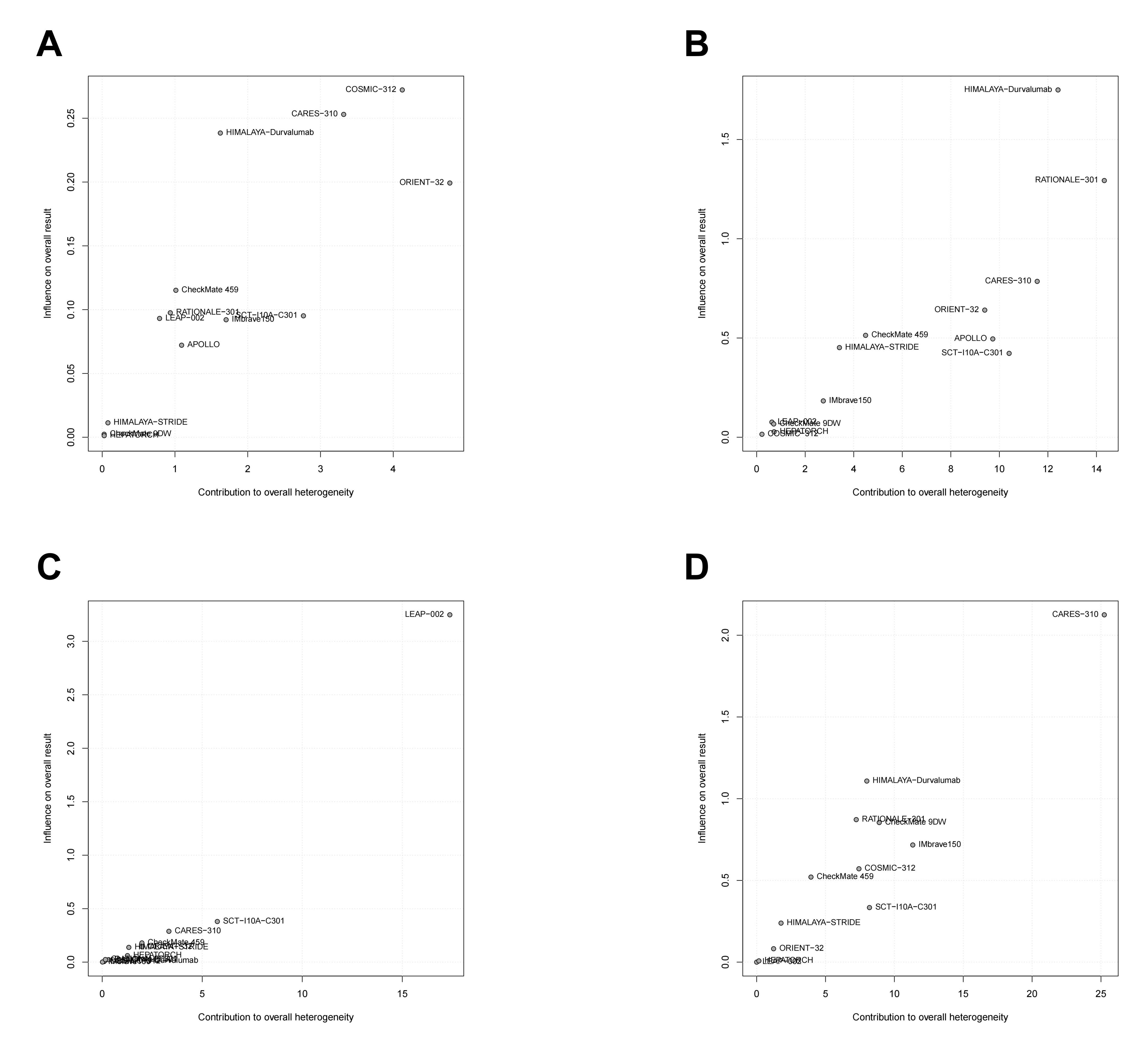


**Supplementary Figure 14.** Baujat plot for detecting publication bias. A) Baujat plot for OS; B) Baujat plot for PFS; C) Baujat plot for ORR; D) Baujat plot for DCR.





**Supplementary Figure 15.** Forest plots of sensitivity analysis for meta-analysis. A) Forest plot for OS using fixed effect model; B) Forest plot for OS using random effect model; C) Forest plot for PFS using fixed effect model; D) Forest plot for PFS using random effect model; E) Forest plot for ORR using fixed effect model; F) Forest plot for ORR using random effect model; G) Forest plot for DCR using fixed effect model; H) Forest plot for DCR using random effect model.
